# Supplementary material for: RNA-induced PRC2 inhibition depends on the sequence of bound RNA
Source: Nat Commun. 2026 Apr 23;17:5605. doi: 10.1038/s41467-026-72294-y (PMC13316113; doi:10.1038/s41467-026-72294-y)
Supplement: Supplementary file 1 — Supplementary Information [file 41467_2026_72294_MOESM1_ESM.pdf]

# Supplementary Information

# RNA-induced PRC2 inhibition depends on the sequence of bound RNA

Jiarui Song[1,2,3], Liqi Yao[1], Anne R. Gooding[1,2,3], Valentin Thron[1,2,3,4], Wayne O. Hemphill[1,2,3], Karen J. Goodrich[1,2,3], Annette H. Erbse[1], Vignesh Kasinath[1]* and Thomas R. Cech[1,2,3]*

[1]Department of Biochemistry, University of Colorado Boulder, Boulder, CO 80303, USA.

[2]BioFrontiers Institute, University of Colorado Boulder, Boulder, CO 80303, USA.

[3]Howard Hughes Medical Institute, University of Colorado Boulder, Boulder, CO 80303, USA.

[4]Present address: University of Regensburg, 93053 Regensburg, Germany

*Corresponding authors. Email: thomas.cech@colorado.edu (T.R.C.); vignesh@colorado.edu (V.K.)

**The PDF file includes:**

Supplementary Figures 1-10

Supplementary Table 1

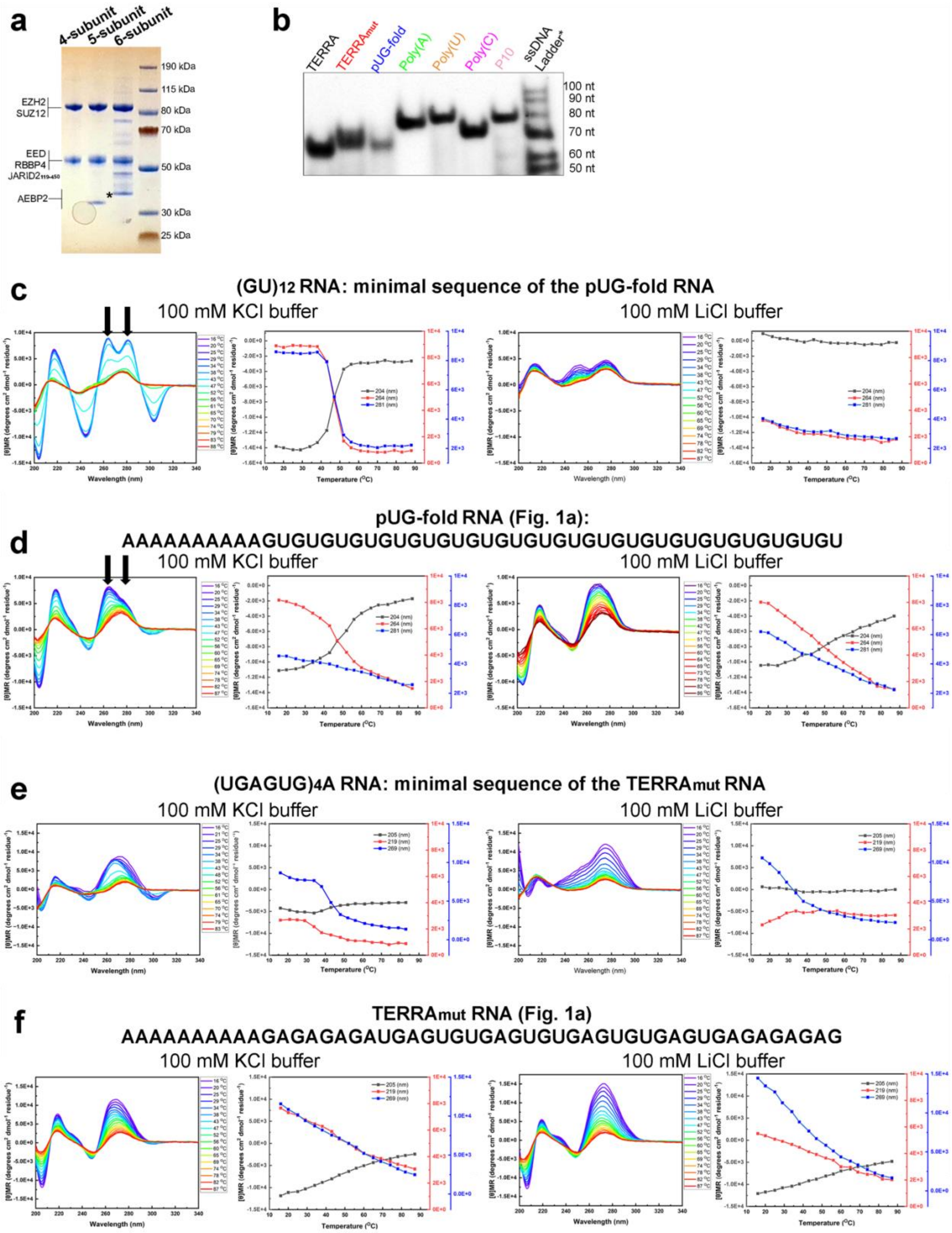
a
4-subunit
5-subunit
6-subunit
190 kDa
115 kDa
80 kDa
70 kDa
50 kDa
30 kDa
25 kDa
EZH2
SUZ12
EED
RBBP4
JARID2119-450
AEBP2
*
b
TERRA
TERRAmut
pUG-fold
Poly(A)
Poly(U)
Poly(C)
P10
ssDNA Ladder*
100 nt
90 nt
80 nt
70 nt
60 nt
50 nt
c
(GU)12 RNA: minimal sequence of the pUG-fold RNA
100 mM KCl buffer
100 mM LiCl buffer
Wavelength (nm)
Temperature (°C)
d
pUG-fold RNA (Fig. 1a):
AAAAAAAAAAGUGUGUGUGUGUGUGUGUGUGUGUGUGUGUGUGUGUGU
100 mM KCl buffer
100 mM LiCl buffer
e
(UGAGUG)4A RNA: minimal sequence of the TERRAmut RNA
100 mM KCl buffer
100 mM LiCl buffer
f
TERRAmut RNA (Fig. 1a)
AAAAAAAAAAGAGAGAGAUGAGUGUGAGUGUGAGUGUGAGUGAGAGAGAG
100 mM KCl buffer
100 mM LiCl buffer

**Supplementary Fig. 1: Purified PRC2 complexes and structural analysis of RNA oligonucleotides used in this study.** (a) Coomassie-stained SDS polyacrylamide gel of purified four-, five-, and six-subunit PRC2 complexes. *AEBP2 of the six-subunit PRC2 has seven additional amino acids at N-terminal after tag cleavage, which is unstructured flexible linker without changing AEBP2 function. (b) Native gel electrophoresis of RNA. RNAs were folded in $K^+$ buffer and then analyzed using a native polyacrylamide gel run in $K^+$-containing buffer. *The size marker in this assay was single-stranded DNA ladder (IDT, 20/100 Ladder) which is not an accurate size-reference for RNAs, only serving as a reference for reproducibility. (c-f) Circular Dichroism (CD) melt assay showing the temperature dependence of CD spectra and corresponding melting curves at the indicated wavelengths for RNAs in 100 mM KCl buffer (left) or 100 mM LiCl buffer (right). (c) $(GU)_{12}$ is the minimal sequence requirement for RNA to adopt the pUG-fold structure, characterized by a distinctive double-peak feature between 260 and 290 nm in the CD spectrum (black arrows). This signature is $K^+$-dependent, as the same RNA in LiCl buffer lacks this feature. (d) pUG-fold RNA used in other assays in this study (Fig. 1a) shows the same $K^+$-dependent signature. Although inclusion of the non-structured $(A)_{10}$ linker reduces the prominence of the double peak, it remains detectable in KCl buffer and is absent in LiCl buffer. (e-f) $(UGAGUG)_4A$ and $TERRA_{mut}$ RNAs, designed to inhibit G-quadruplex formation, have CD spectra with neither the double-peak characteristic of the pUG-fold nor the positive peak at 200 nm that is the most reliable feature of G4 structures. Notably, $TERRA_{mut}$ RNA yields near identical spectra and melting behavior in KCl and LiCl buffers.

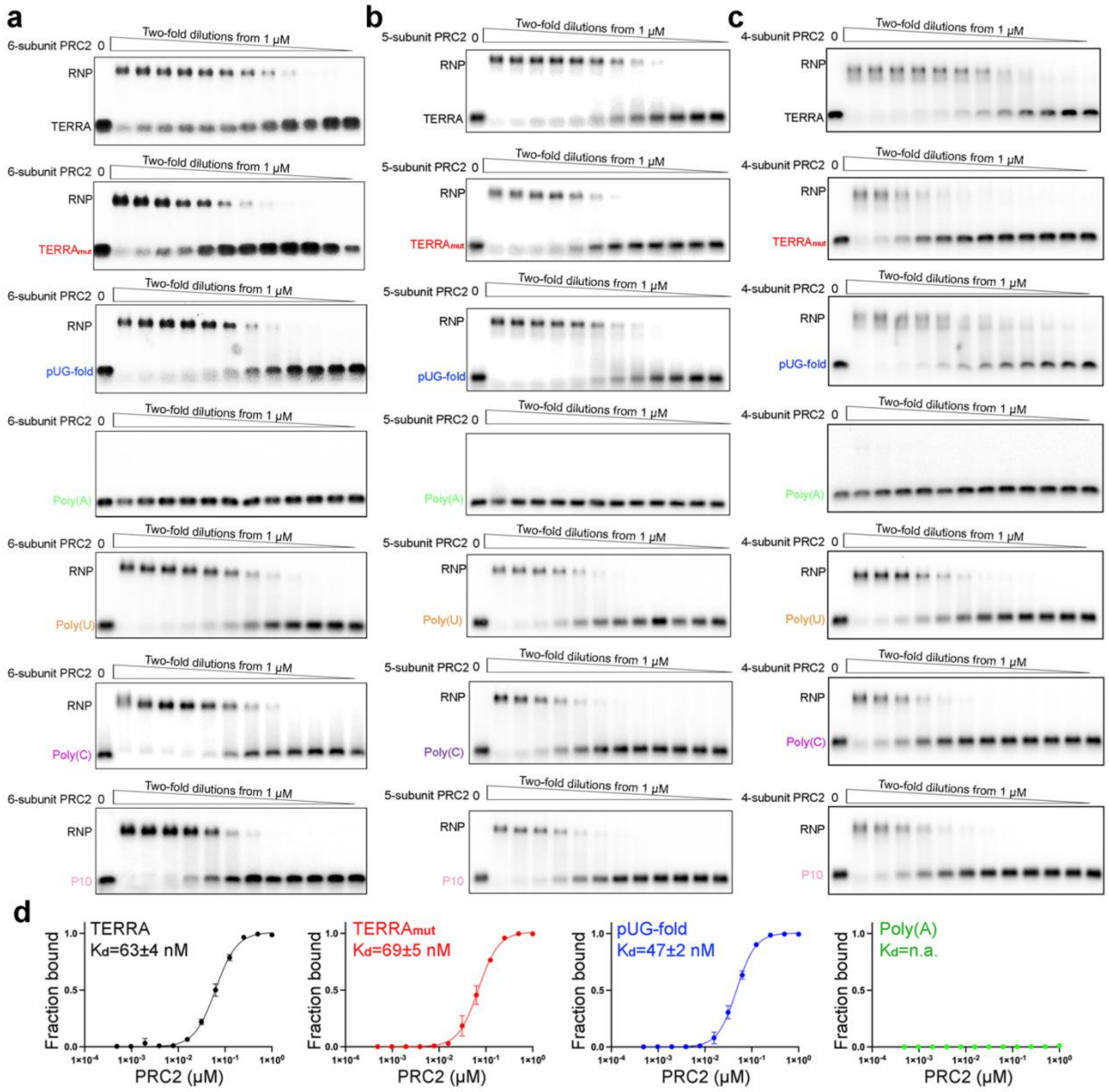


**Supplementary Fig. 2: Six-, five- and four-subunit PRC2 complexes bind RNAs of various sequences and structures.** (a-c) Representative EMSA gels of RNAs binding to the different PRC2 complexes in $K^+$ binding buffer. At least three independent experiments were performed with the same result ($n_{\text{6-subunit PRC2}}=6$, $n_{\text{5-subunit PRC2}}=3$, $n_{\text{4-subunit PRC2 and TERRAmut RNA}}=6$, and $n_{\text{4-subunit PRC2 and other RNAs}}=3$) (d) Quantification of EMSA results in a $Li^+$ binding buffer in which the G-quadruplexes are not folded. The binding affinity of TERRA is reduced to the same affinity as TERRA$_{mut}$ RNA. Two independent experiments were performed with the same result (n=2). Symbols indicate the average of two replicates with individual values shown as bars.

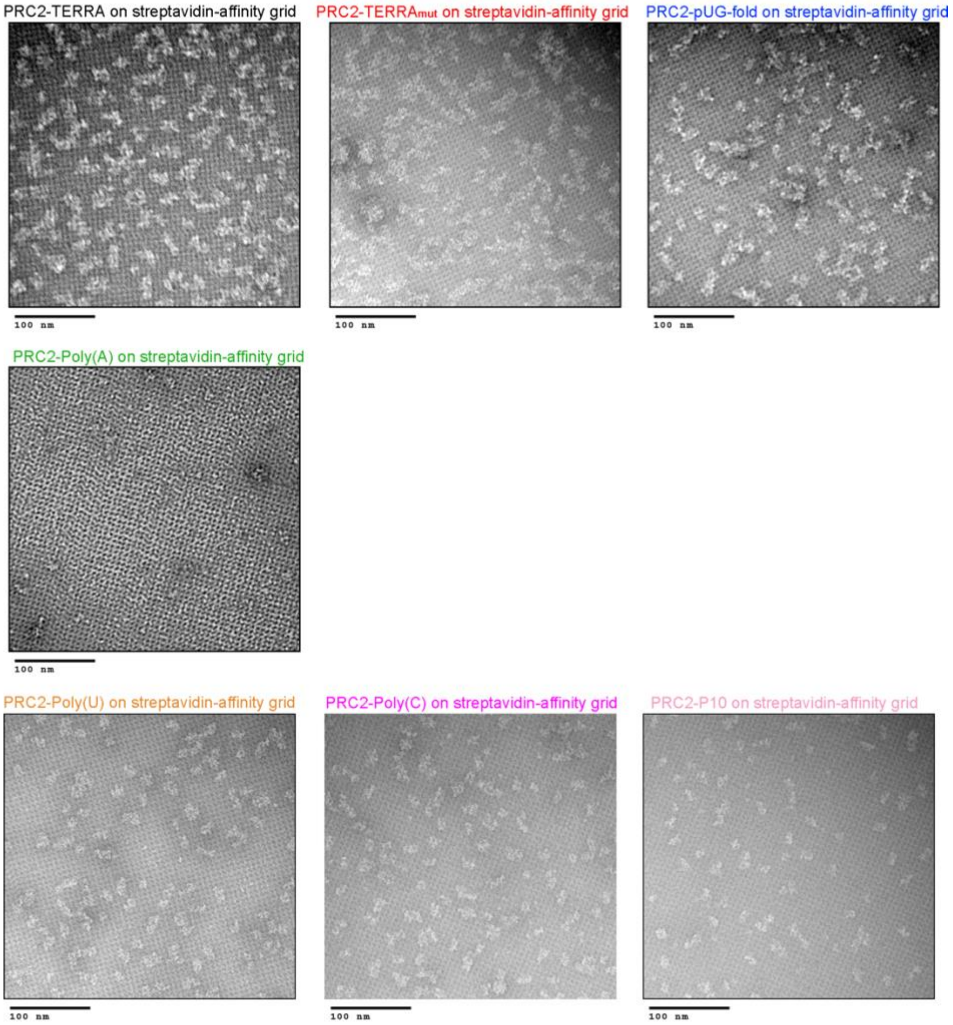


**Supplementary Fig. 3: Streptavidin-affinity EM method selects for RNA-bound PRC2 six-subunit complex.** Representative negative staining EM images of PRC2-TERRA, PRC2-$TERRA_{mut}$, PRC2-pUG-fold, PRC2-Poly(A), PRC2-Poly(U), PRC2-Poly(C), and PRC2-P10. All RNAs are biotinylated. Poly(A) does not bind PRC2, and therefore, few recognizable particles are observed in the image. The streptavidin lattice is clearly visible in the background of all images.

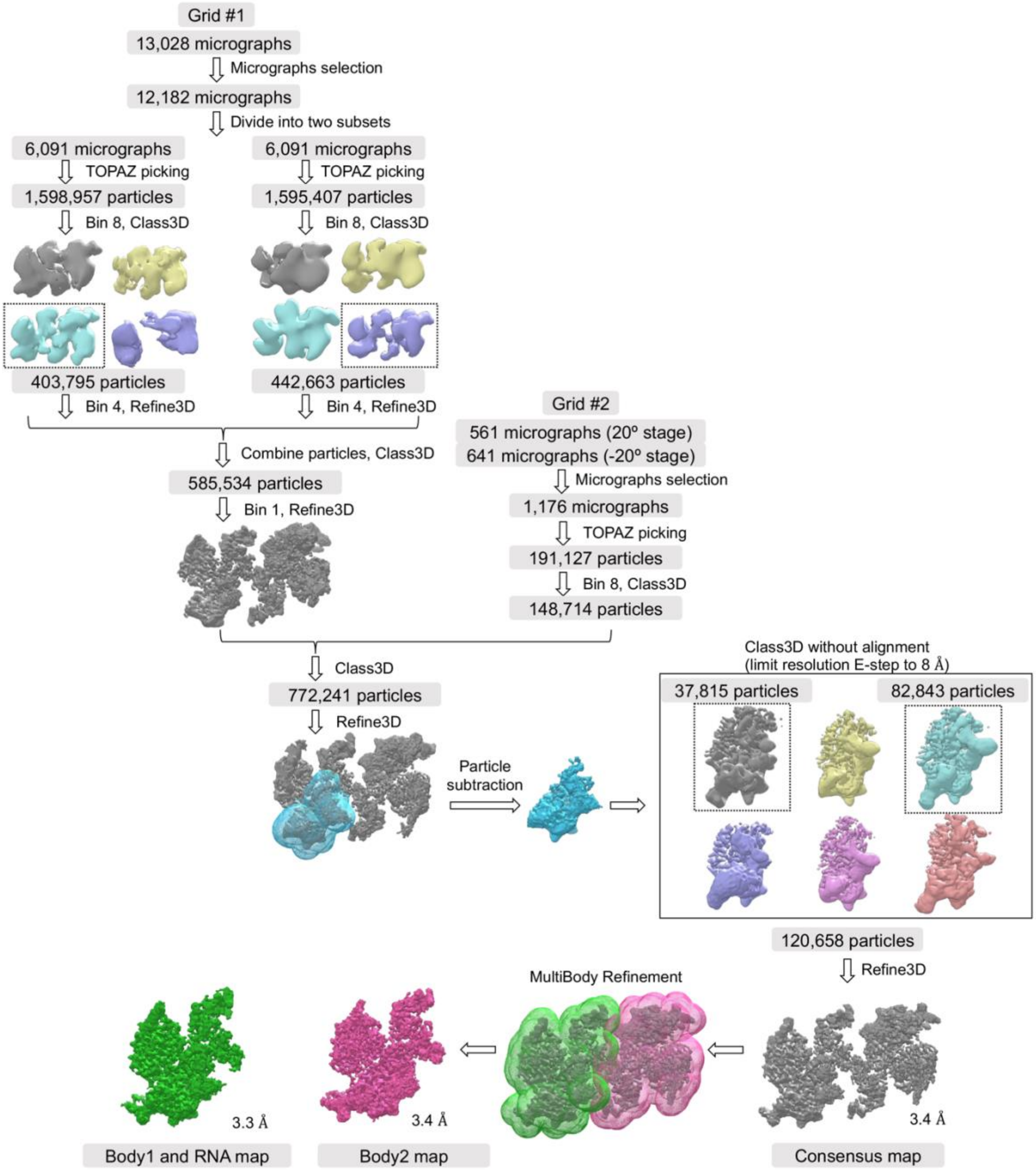


**Supplementary Fig. 4: Single-particle cryo-EM image processing workflows for PRC2-TERRA$_{mut}$ RNA complex.**

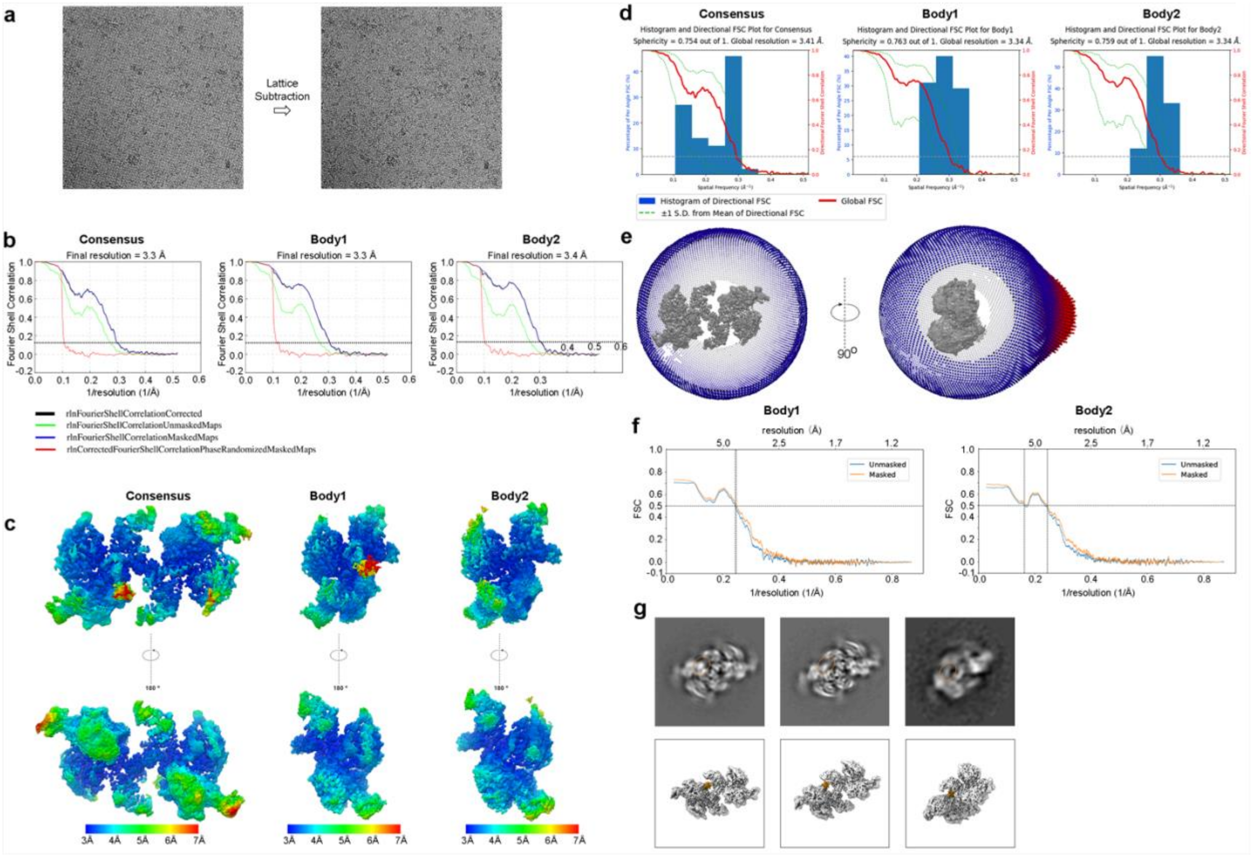


**Supplementary Fig. 5: Cryo-EM map analysis.** (a) Cryo-EM micrographs before and after streptavidin lattice subtraction. (b) Fourier shell correlation (FSC) curves of consensus map and maps of two individual bodies from multibody refinement. 0.143 intercepts are indicated by dashed lines. (c) Local-resolution density maps of consensus and two bodies. (d) 3D FSC of consensus map and two bodies. (e) Euler angle distribution for the particles after consensus map refinement. (f) Model vs Map FSC for Phenix-refined models of two bodies. (g) Top: A subset of 2D class averages from the final particles used to generate the consensus map. Bottom: 3D density maps at the corresponding orientations. $TERRA_{mut}$ RNA density is highlighted by orange circles in 2D averages and orange color in 3D maps, respectively.

# AlphaFold 3 predictions of PRC2-TERRAmut RNA

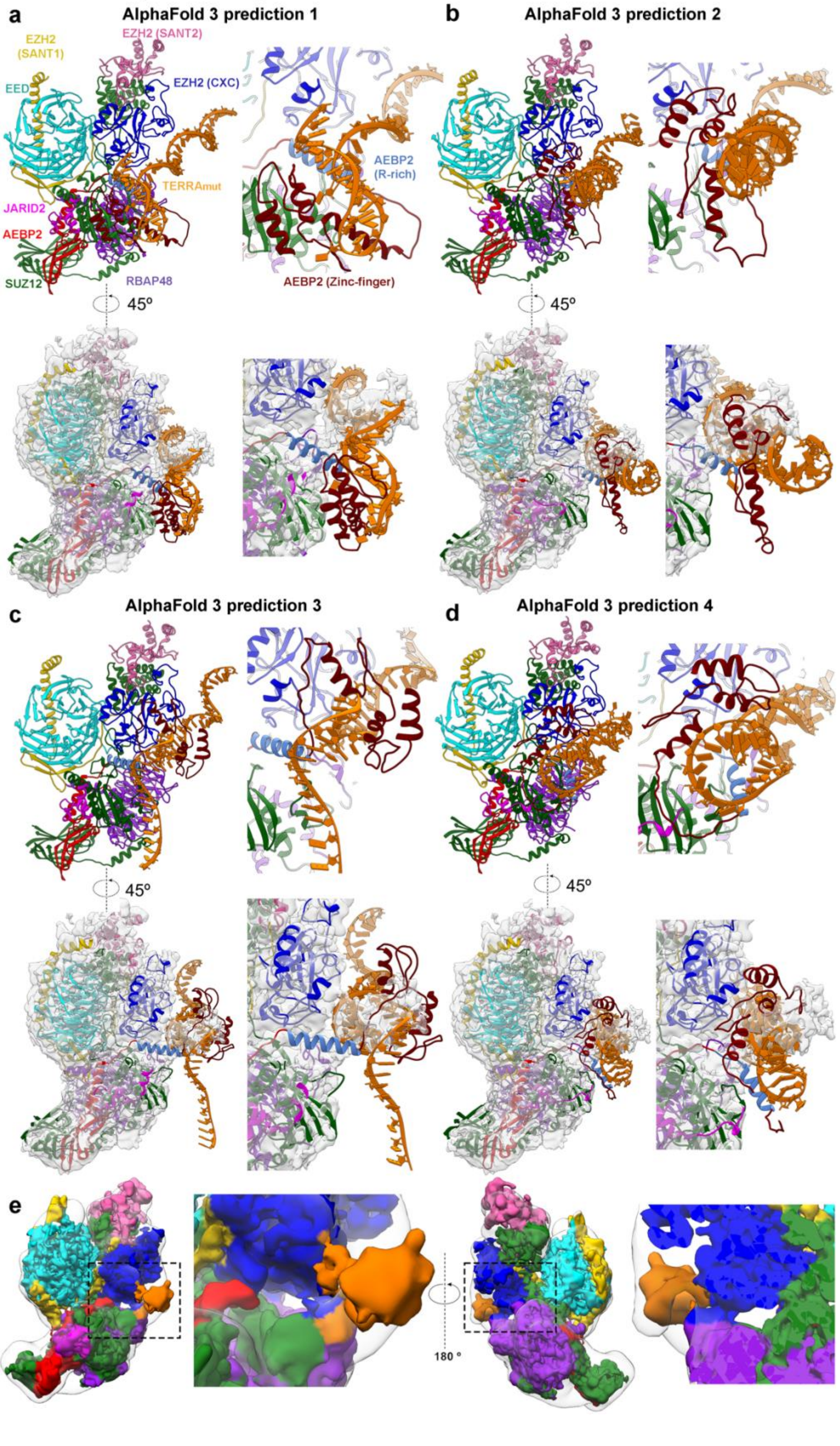

**Supplementary Fig. 6: AlphaFold 3 generated predictions of the $TERRA_{mut}$ RNA-single PRC2 complex.** (a) Top left: Model from AlphaFold 3 prediction. Top right: Close-up view of $TERRA_{mut}$ RNA and peripheral protein regions. Bottom left: Model is overlapped onto the cryo-EM density to emphasize the intrinsic flexibility of PRC2-$TERRA_{mut}$ binding. Bottom right: Zoom-in view. (b-d) Three additional AlphaFold 3 predictions displayed as in panel a. (e) Local resolution filtered cryo-EM map of $TERRA_{mut}$ RNA-bound PRC2 protomer (colored) is overlapped with the density map generated from only the overlapping regions of the top four AlphaFold 3 predictions shown above (transparent). Dashed boxes are zoomed in to present details.

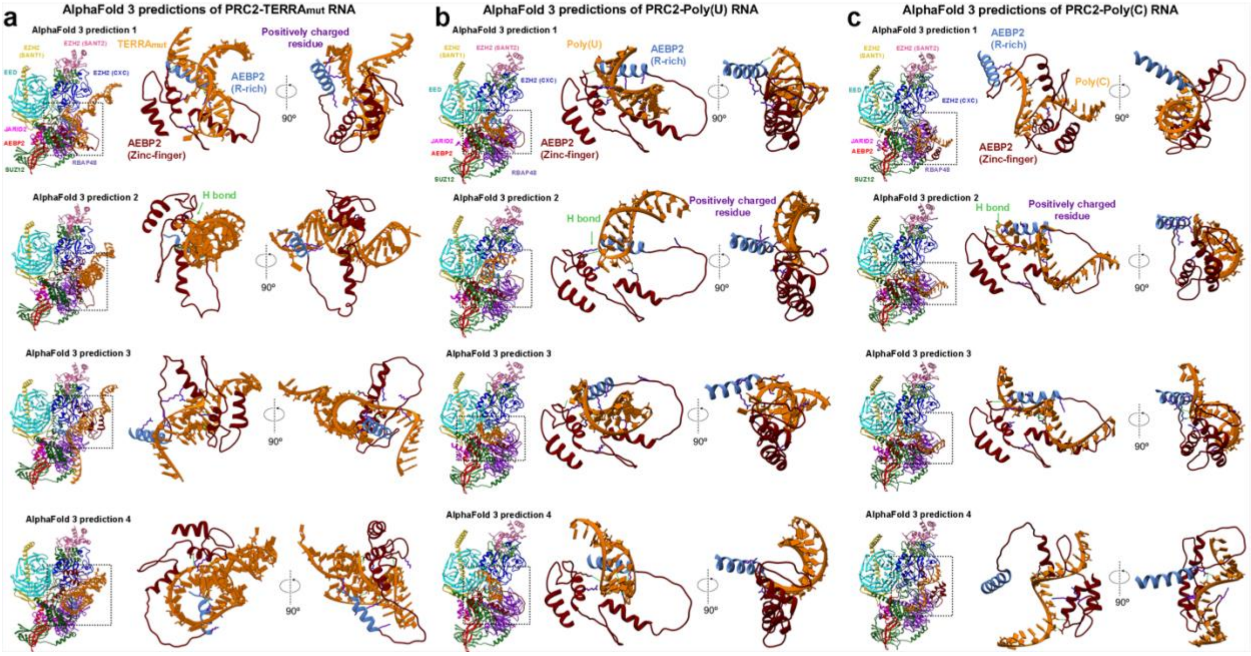


**Supplementary Fig. 7: AlphaFold 3 predicted interactions between AEBP2 and RNA.** (a-c) Left: Overall models from AlphaFold 3 predictions. Dashed boxes are zoomed in. Center and Right: Zoom-in views of RNA, AEBP2 arginine-rich segment, and zinc-finger domains. Hydrogen bonds were analyzed by the FindHbond function built in Chimera and highlighted in green color. Side chains of positively charged residues aiming towards RNA are also highlighted in purple color.

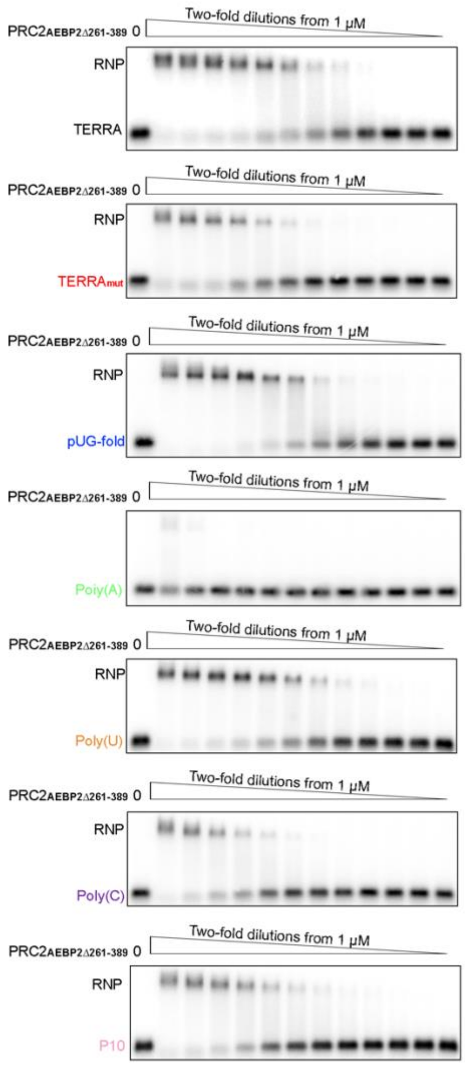


**Supplementary Fig. 8: PRC2$_{AEBP2Δ261-389}$ mutant has decreased binding towards TERRA$_{mut}$ and Poly(C).** Representative EMSA gels. Three independent experiments were performed with the same result (n=3). Quantification of EMSA results is shown in Fig. 4b.

**a** **Chimeric RNA** (P10-TERRA):
5’ CUCUUUCUCUCCUUCUCUUC(A)**10**(UUAGGG)**4** 3’

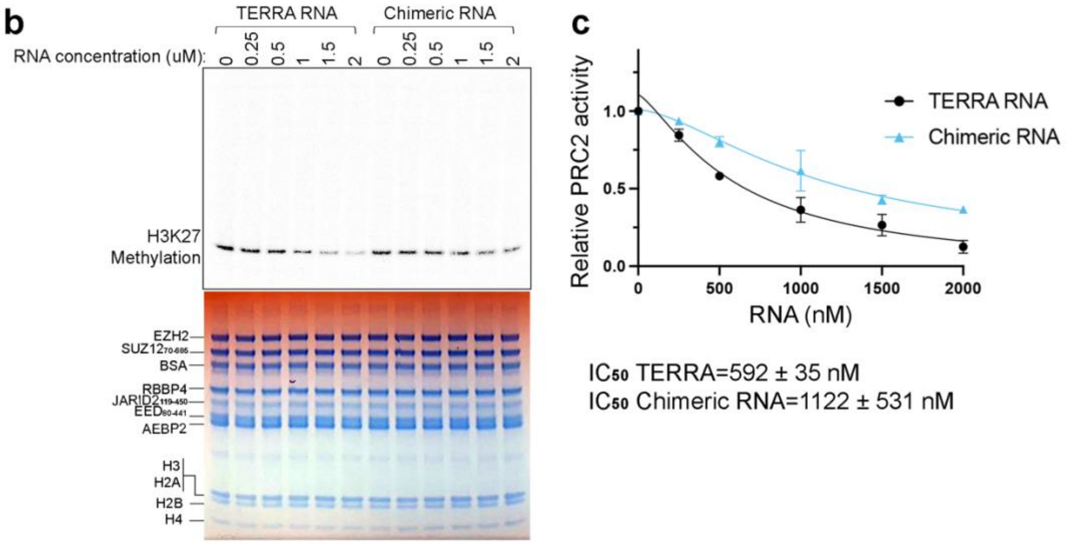


**Supplementary Fig. 9: RNA sequence that induces PRC2 dimerization and inhibition is the dominant element in a chimeric RNA.** (a) Sequences of the chimeric RNA used in this assay. It has a 5’ 20-nucleotide P10 sequence, a 10-nucleotide poly(A) linker, and a 3’ complete TERRA. (b) Representative histone methyltransferase activity assays with $^{14}C$-labeled S-adenosylmethionine analyzed by SDS-PAGE, with gels imaged for $^{14}C$ signals (top) or stained with Coomassie blue to confirm equal loading of PRC2 and nucleosomes (bottom). Three independent experiments were performed with the same result (n=3). (c) Quantification of three replicates. Error bars are mean ± standard deviation. $IC_{50}$ is the concentration of RNA that inhibits 50% of PRC2 activity relative to no RNA.

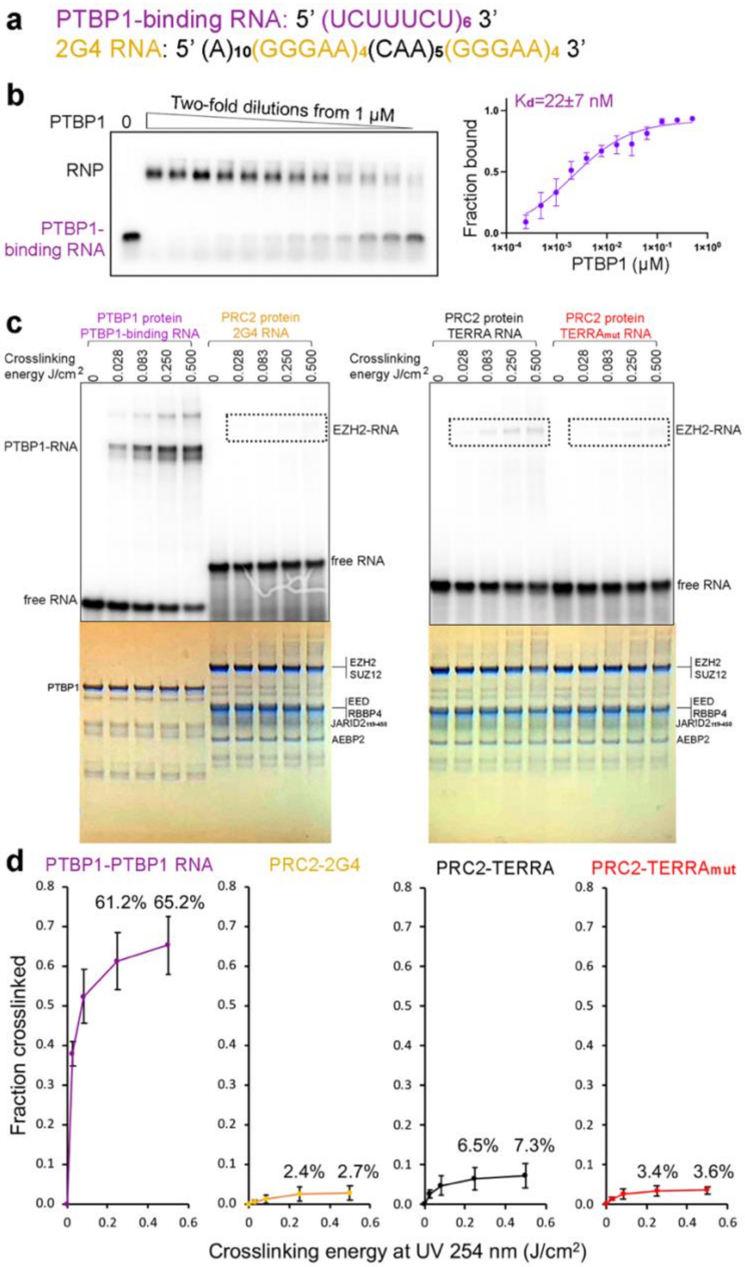


**Supplementary Fig. 10: UV crosslinking has very low efficiency for capturing PRC2-RNA associations.** (a) Sequences of RNA oligos used in this assay. (b) EMSA result of recombinant PTBP1 binding to $(UCUUUCU)_6$ RNA. Left: representative EMSA gel. Two independent experiments were performed with the same result (n=2). Right: quantification of EMSA results. Symbols indicate the average of two replicates with individual values shown as bars. (c) Representative UV crosslinking assays. In all reactions, proteins were 0.6 μM (at least 10-times $K_d$) to ensure saturated RNA-binding. Gels were imaged to reveal crosslinking of radiolabeled RNA (top) or stained with Coomassie blue to confirm equal loading of proteins (bottom). Dashed boxes are positions expected for crosslinked EZH2-RNA complexes. Three independent experiments were performed with the same result (n=3). (d) Quantification of three UV crosslinking replicates. Error bars are mean ± standard deviation.

**Table S1. Cryo-EM data collection and refinement statistics**

| | Multibody body 1 | Multibody body 2 | Consensus map |
|---|---|---|---|
| Data collection and processing | | | |
| Magnification | 130,000 | 130,000 | 130,000 |
| Voltage (kV) | 300 | 300 | 300 |
| Camera | Falcon 4 | Falcon 4 | Falcon 4 |
| Electron exposure ($e^-/Å^2$) | 50 | 50 | 50 |
| Exposure rate (e-/pixel*s) | 8.78 | 8.78 | 8.78 |
| Number of frames | 1323 | 1323 | 1323 |
| Defocus range (μm) | -0.5 to -1.9 | -0.5 to -1.9 | -0.5 to -1.9 |
| Pixel size (Å) | 0.97 | 0.97 | 0.97 |
| Symmetry imposed | C1 | C1 | C1 |
| Movies collected (no.) | 13,028 + 1202 (tilted stage) | 13,028 + 1202 (tilted stage) | 13,028 + 1202 (tilted stage) |
| Initial particle images (no.) | 3,194,364 + 191,127 (tilted stage) | 3,194,364 + 191,127 (tilted stage) | 3,194,364 + 191,127 (tilted stage) |
| Final particle images (no.) | 105,974 + 14,684 (tilted stage) | 105,974 + 14,684 (tilted stage) | 105,974 + 14,684 (tilted stage) |
| Map resolution (Å) | | | |
| FSC0.143 (unmasked/masked) | 3.8/3.3 | 3.8/3.4 | 3.7/3.4 |
| Map resolution range (Å) | 3-10 | 3-11 | 3-11 |
| Refinement | | | |
| Initial model used (PDB code) | 8FYH | 8FYH | 8FYH |
| Resolution cutoff (Å) | 4 | 4 | 4 |
| Map sharpening *B* factor ($Å^2$) | 0 | 0 | 0 |
| Model composition | | | |
| Non-hydrogen atoms | 12111 | 12103 | 24433 |
| Protein residues | 1793 | 1793 | 3586 |
| Nucleotide residues | 0 | 0 | 10 |
| Ligands | ZN:7 | ZN:7 | ZN:14 |
| *B* factors ($Å^2$) | | | |
| Protein | 24.35 | 13.42 | 13.42 |
| Ligand | 44.27 | 22.70 | 22.70 |
| RMSD | | | |
| Bond lengths (Å) | 0.002 (0) | 0.002 (0) | 0.004 (0) |
| Bond angles (°) | 0.521 (4) | 0.536 (6) | 0.971 (4) |
| Validation | | | |
| MolProbity score | 2.25 | 2.27 | 2.26 |
| Clashscore | 9.77 | 8.81 | 9.57 |
| Poor rotamers (%) | 2.01 | 2.58 | 2.18 |
| Ramachandran plot | | | |
| Favored (%) | 91.43 | 92.12 | 91.66 |
| Allowed (%) | 8.45 | 7.77 | 8.17 |
| Disallowed (%) | 0.11 | 0.11 | 0.17 |
