# Supplementary material for: RNA-induced PRC2 inhibition depends on the sequence of bound RNA
Source: Nat Commun. 2026 Apr 23;17:5605. doi: 10.1038/s41467-026-72294-y (PMC13316113; doi:10.1038/s41467-026-72294-y)
Supplement: Supplementary file 2 — Reporting Summary [file 41467_2026_72294_MOESM2_ESM.pdf]

nature portfolio

Corresponding author(s): Vignesh Kasinath
Thomas R. Cech

Last updated by author(s): Mar 24, 2026

Reporting Summary

Nature Portfolio wishes to improve the reproducibility of the work that we publish. This form provides structure for consistency and transparency in reporting. For further information on Nature Portfolio policies, see our Editorial Policies and the Editorial Policy Checklist.

Statistics

For all statistical analyses, confirm that the following items are present in the figure legend, table legend, main text, or Methods section.

n/a	Confirmed	
☐	☒	The exact sample size (*n*) for each experimental group/condition, given as a discrete number and unit of measurement
☐	☒	A statement on whether measurements were taken from distinct samples or whether the same sample was measured repeatedly
☐	☒	The statistical test(s) used AND whether they are one- or two-sided<br>*Only common tests should be described solely by name; describe more complex techniques in the Methods section.*
☒	☐	A description of all covariates tested
☒	☐	A description of any assumptions or corrections, such as tests of normality and adjustment for multiple comparisons
☐	☒	A full description of the statistical parameters including central tendency (e.g. means) or other basic estimates (e.g. regression coefficient) AND variation (e.g. standard deviation) or associated estimates of uncertainty (e.g. confidence intervals)
☒	☐	For null hypothesis testing, the test statistic (e.g. *F*, *t*, *r*) with confidence intervals, effect sizes, degrees of freedom and *P* value noted<br>*Give P values as exact values whenever suitable.*
☒	☐	For Bayesian analysis, information on the choice of priors and Markov chain Monte Carlo settings
☒	☐	For hierarchical and complex designs, identification of the appropriate level for tests and full reporting of outcomes
☒	☐	Estimates of effect sizes (e.g. Cohen's *d*, Pearson's *r*), indicating how they were calculated

Our web collection on statistics for biologists contains articles on many of the points above.

Software and code

Policy information about availability of computer code

Data collection	Smart EPU Cryo-EM collection software, ThermoFisher Scientific
Data analysis	RELION 5.0.0 RRID:SCR_016274<br>MotionCor2 RRID:SCR_016499<br>CTFFIND 4 4.1.14 RRID:SCR_016732<br>CryoSPARC v5.0.3 RRID:SCR_016501<br>COOT 0.9.8.8 RRID:SCR_014222<br>PHENIX 1.4 RRID:SCR_014224<br>UCSF Chimera 1.17.1 RRID:SCR_004097<br>UCSF ChimeraX 1.11 RRID:SCR_015872<br>AlphaFold 3 Server RRID:SCR_025885<br>MATLAB R2025b RRID:SCR_001622<br>ImageQuantTL 10.2.499 RRID:SCR_014246<br>Prism 10 10.2.3 RRID:SCR_002798

For manuscripts utilizing custom algorithms or software that are central to the research but not yet described in published literature, software must be made available to editors and reviewers. We strongly encourage code deposition in a community repository (e.g. GitHub). See the Nature Portfolio guidelines for submitting code & software for further information.

Data

Policy information about availability of data

All manuscripts must include a data availability statement. This statement should provide the following information, where applicable:

- Accession codes, unique identifiers, or web links for publicly available datasets
- A description of any restrictions on data availability
- For clinical datasets or third party data, please ensure that the statement adheres to our policy

Cryo-EM density maps and fitted models have been deposited in the Electron Microscopy Data Bank (EMD-46751, consensus map; EMD-46722, Body1 from multibody refinement; and EMD-46726, Body2 from multibody refinement) and the Protein Data Bank (PDB: 9DCH). Source data are deposited in Figshare (DOI: 10.6084/m9.figshare.30456503) and provided with this paper. Requests for reagents, plasmids and cell lines used in this study should be directed to the corresponding authors.

Research involving human participants, their data, or biological material

Policy information about studies with human participants or human data. See also policy information about sex, gender (identity/presentation), and sexual orientation and race, ethnicity and racism.

Reporting on sex and gender	This study does not involve human participants so no such information is collected.
Reporting on race, ethnicity, or other socially relevant groupings	N.A.
Population characteristics	N.A.
Recruitment	N.A.
Ethics oversight	N.A.

Note that full information on the approval of the study protocol must also be provided in the manuscript.

Field-specific reporting

Please select the one below that is the best fit for your research. If you are not sure, read the appropriate sections before making your selection.

☒ Life sciences ☐ Behavioural & social sciences ☐ Ecological, evolutionary & environmental sciences

For a reference copy of the document with all sections, see nature.com/documents/nr-reporting-summary-flat.pdf

Life sciences study design

All studies must disclose on these points even when the disclosure is negative.

Sample size	No formal sample size calculation was performed. Sample sizes were selected on the basis of standard practice in the field, sample availability, and the need to ensure reproducibility across independent experiments. For biochemical and biophysical assays, at least three independent replicates were performed unless otherwise stated, and the exact n values are provided in the corresponding figures or figure legends. These sample sizes were sufficient to support the conclusions because independent experiments yielded consistent results. For EM studies, the amount of data collected are provided in the corresponding figures, figure legends or methods.
Data exclusions	No data were excluded from analysis.
Replication	For each experiment, detailed description of number of replicates, sample size and statistics is provided in figure legend and methods.
Randomization	The samples for each experiment were randomized to be examined (No specific methods were used).
Blinding	The investigator was not blinded. The experiments in this study are in vitro biochemical, biophysical and structural analyses performed on predefined samples with known composition, and the outcomes were determined using objective measurements.

Reporting for specific materials, systems and methods

We require information from authors about some types of materials, experimental systems and methods used in many studies. Here, indicate whether each material, system or method listed is relevant to your study. If you are not sure if a list item applies to your research, read the appropriate section before selecting a response.

Materials & experimental systems

n/a	Involved in the study
☒	☐ Antibodies
☐	☒ Eukaryotic cell lines
☒	☐ Palaeontology and archaeology
☒	☐ Animals and other organisms
☒	☐ Clinical data
☒	☐ Dual use research of concern
☒	☐ Plants

Methods

n/a	Involved in the study
☒	☐ ChIP-seq
☒	☐ Flow cytometry
☒	☐ MRI-based neuroimaging

Eukaryotic cell lines

Policy information about cell lines and Sex and Gender in Research

Cell line source(s)	Sf9 cells (Spodoptera frugiperda, IPLB-Sf-21-AE) were obtained from ThermoFisher (LOT:11496015) and maintained in Sf-900 III SFM medium (ThermoFisher:12658019) supplemented with Antibiotic-Antimycotic (Millipore Sigma: A5955) at 27 degree. High Five cells (Trichoplusia ni, BTI-TN-5B1-4) were obtained from ThermoFisher (LOT: B85502) and maintained in ESF 921 Insect Cell Culture Medium (Expression System: 96-001-01) supplemented with Antibiotic-Antimycotic ((Millipore Sigma: A5955) at 27 degree.
Authentication	Cell lines were verified by manufacturer's website
Mycoplasma contamination	Cells were routinely tested for mycoplasma contamination by the University of Colorado Boulder Cell Culture Facility (RRID:SCR_018988).
Commonly misidentified lines (See ICLAC register)	No cell lines used in this study were found in the database of commonly misidentified cell lines

Plants

Seed stocks	N.A.
Novel plant genotypes	N.A.
Authentication	N.A.
