# Supplementary material for: RNA-induced PRC2 inhibition depends on the sequence of bound RNA
Source: Nat Commun. 2026 Apr 23;17:5605. doi: 10.1038/s41467-026-72294-y (PMC13316113; doi:10.1038/s41467-026-72294-y)
Supplement: Supplementary file 3 — Transparent Peer Review file [file 41467_2026_72294_MOESM3_ESM.pdf]

nature portfolio

Peer Review File

RNA-induced PRC2 inhibition depends on the sequence of bound RNA

Corresponding Author: Dr Thomas Cech

This file contains all reviewer reports in order by version, followed by all author rebuttals in order by version.

Attachments originally included by the reviewers as part of their assessment can be found at the end of this file.

Version 0:

Reviewer comments:

Reviewer #1

(Remarks to the Author)
In this manuscript Song et al. continue their work on structurally and mechanically characterising the regulation of the histone methyltransferase complex PRC2 by the interaction with RNAs. In their foundational paper [Song et al. 2023] they had described the structural basis of PRC2 regulation by G-quadruplex RNAs. Many of the RNAs PRC2 has been reported to interact with have however no canonical G-quadruplex sequences and/or structures. To address this conundrum the authors systematically test the interactions of a diverse set of RNA sequences with PRC2 using biochemical and structural methods (single particle (cryo)EM but also AlphaFold3 mediated structure prediction).
Their main findings are that PRC2 quiet promiscuously binds to many different RNA sequences and that RNAs without canonical G quadruplex structures can still induce the previously observed inhibitory PRC2 dimer formation. They also show conclusively that only RNA sequences which induce this PRC2 dimer have an inhibitory effect on its histone methyltransferase activity. RNAs that bind PRC2 with high affinity but do not induce dimer formation (e.g. pyrimidine rich sequences) do not inhibit its activity. These insights reconciles previous findings of apparent contradictory effects of different RNA sequences on PRC2 activity.
The study is thorough in its inquiry and the presented data, especially the EM data, are beautifully displayed, conclusive and carefully interpreted.

Minor concerns:
In the previously reported EM map of the G-quadruplex induced PRC2 dimer (EMD-29578; PDB:8FYH) the G-quadruplex appears to contact both PRC2 promoters. It is unclear to this reviewer wether this is also the case with their TERRAmut RNA here. Do the authors observe unexplained density which might be RNA related? If not is this potentially be an issue of a too tight map around the RNA density?

Reviewer #2

(Remarks to the Author)
In this manuscript, Song et al. investigate how RNA sequence composition differentially regulates the activity of Polycomb Repressive Complex 2 (PRC2). Through a combination of biochemical assays, negative-stain and cryo-electron microscopy, AlphaFold3 modeling, and a CRISPR-dCas9–based RNA localization system, the authors demonstrate that RNA binding to PRC2 is functionally heterogeneous. Specifically, G-rich RNAs, including non-canonical sequences incapable of forming classical G-quadruplexes, induce PRC2 dimerization (AEBP2 and JARID2 included) and inhibit methyltransferase activity, whereas pyrimidine-rich RNAs bind PRC2 in a monomeric, catalytically competent state. The work provides a coherent mechanistic explanation reconciling disparate reports on PRC2-RNA interactions.

The work is technically strong, conceptually interesting, and timely. The authors convincingly demonstrate that PRC2 engages distinct classes of RNAs through mechanistically separable modes of interaction. It represents a substantial advance in our understanding of PRC2–RNA regulation and provides a coherent mechanistic framework that reconciles long-standing inconsistencies in the field. However, in light of the ongoing controversy surrounding PRC2–RNA interactions in cellular contexts, several aspects, particularly the scope and validation of the CRISPR-dCas9–based RNA localization assay, require clearer experimental demonstration to strengthen the manuscript for Nature Communications.

Major Comments

1. Requirement for solid validation using CRISPR-display, related to Figure 5

Although TERRAmut robustly induces PRC2 dimerization and inhibits catalytic activity in vitro, it does not elicit derepression of CDKN2A in the CRISPR-dCas9-based RNA localization assay. While the authors propose plausible explanations, such as reduced effective affinity or competition with endogenous RNA-binding proteins, this discrepancy underscores a key limitation of this cellular assay.
Given the growing number of recent reports describing context-dependent or divergent PRC2–RNA interactions in cells, the robustness and dynamic range of the CRISPR-dCas9-based RNA localization assay must be more clearly established. In this regard, authors should include TERRA WT as a positive control. Demonstrating PRC2 inhibition and target gene derepression at the CDKN2A locus would substantially strengthen the cellular relevance of the conclusions.

2. In vivo (or ex vivo) relevance of RNA-induced PRC2 dimerization

A central conclusion of the manuscript is that RNA-induced PRC2 dimerization underlies sequence-dependent inhibition of PRC2 activity. While this mechanism is convincingly demonstrated in vitro, its prevalence and relevance ex vivo remain insufficiently established. The CRISPR-dCas9 experiments target a single locus (CDKN2A) in one cancer-derived cell line, making it difficult to assess whether RNA-mediated PRC2 inhibition represents a general regulatory mechanism or a locus-specific effect under artificial conditions. Extending these analyses to additional canonical PRC2 target loci, such as the HOXA cluster, would substantially strengthen the generality of the conclusions.

3. Interplay between RNA and nucleosomal DNA binding at AEBP2 Zn finger and the R-rich motif

The authors show that an R-rich motif within AEBP2 contributes to RNA binding. This region has been previously well characterized as a nucleosomal DNA binding interface (PMIDs: 29681498, 33479123). The manuscript would benefit from a more comprehensive discussion integrating these findings, specifically addressing whether RNA and nucleosomal DNA binding are mutually exclusive, competitive, or context-dependent.
It would be of interest to determine whether pyrimidine-rich RNAs preferentially engage the Zn-finger domain while nucleosomal DNA interacts with the R-rich motif, or vice versa, thereby potentially cooperating to facilitate PRC2 recruitment. This possibility could be readily tested using electrophoretic mobility shift assays (EMSA).

4. Based on the dimerization and PRC2 binding results, it would be informative to assess whether TERRAmut fails to induce dimerization of the four-subunit PRC2 core complex.

Minor Comment

1. In this sentence, "We first prepared the six-subunit PRC2 complex with accessory proteins AEBP2 and JARID2, as these accessory proteins have previously been implicated in RNA binding…", the authors should explicitly state that they used the short (embryonic) isoform of AEBP2 and a truncated form of JARID2. The precise transcript variant and amino-acid boundaries (e.g., aa xx–yy) should be clearly specified for consistency with the Materials and Methods as well.

2. In this sentence, "Thus, our systematic characterizations indicate that the four-subunit holoenzyme contains all essential protein elements required to recognize G4 as the most preferred RNA, consistent with our cryo-EM structure, which shows that the catalytic subunit EZH2 is predominantly responsible for G4 RNA recognition.", the authors should add the appropriate reference supporting the cryo-EM structural evidence.

3. Figure 1a: Including a simplified schematic or cartoon representation of the RNA secondary/tertiary structures would help readers more immediately grasp the rationale behind RNA selection. This does not need the level of detail shown in Extended Data Fig. 1a, but should clearly convey structural differences among the RNAs used.

4. Overall, the manuscript is occasionally difficult to follow, as essential information is dispersed across multiple sections and different PRC2 complexes are used in different experiments. In order to increase the readability, I suggest several points as follows.

(1) Clearly specify, both in the figure legends and at the first mention in the main text, which PRC2 complex was used for each experiment. For example, the authors should explicitly state when the six-subunit PRC2 complex was used for cryo-EM, size-exclusion chromatography, and related analyses.

(2) In the legend of Figure 5a, the authors should mention the cell line they used.

5. Statistical reporting: Exact numbers of replicates (n) should be more consistently reported in figure legends, particularly for biochemical assays.

Reviewer #3

(Remarks to the Author)
Review Comments:

This study by Song et al. investigates the structural and functional consequences of RNA binding to Polycomb Repressive Complex 2 (PRC2). Using Cryo-EM and biochemical assays, the authors identify two distinct binding modes: G-rich RNAs (including non-G4 sequences like TERRAmut) induce PRC2 dimerization and enzymatic inhibition, whereas pyrimidine-rich RNAs bind PRC2 monomers via the AEBP2 subunit without inhibiting activity. The authors attempt to extend these findings to a cellular context using a CRISPR-dCas9 display system. This work addresses a long-standing debate regarding the specificity and outcome of PRC2-RNA interactions, offering a potentially unifying structural model where functional outcomes (inhibition vs. recruitment) are sequence-dependent. However, a significant gap remains between the rigorous in vitro structural data and the in vivo functional assays. Specifically, the finding that TERRAmut induces potent inhibition in vitro but is completely inactive in cells (Fig. 5) challenges the physiological relevance of the proposed non-G4 dimerization mechanism. Furthermore, the exclusion of standard in vivo binding validation (based on the inefficiency of UV crosslinking shown in Extended Data Fig. 10) leaves the cellular mechanism unproven. While the structural insights are novel, substantial revision including rigorous biophysical validation and more robust cellular evidence is required to substantiate the biological conclusions to meet the need for the scope of publication in Nature Communications.

Major concerns:
1. While the in vitro characterization is extensive, the in vivo evidence is sparse, relying heavily on the functional outcome of the CRISPR-dCas9 system without validating the intermediate steps. There is a notable contrast between the results in Figure 4b, where TERRAmut significantly inhibits PRC2, and Figure 5, where this inhibitory effect is abolished in cells. The authors attribute this to "lower binding affinity," but this remains speculative. To support the mechanistic conclusions within a reasonable revision timeline, the following are necessary:
(1) The authors argue in Extended Data Fig. 10 that UV crosslinking is inefficient for PRC2. However, the lack of in vivo binding data leaves a gap in the mechanism. To confirm that the dCas9-tethered TERRAmut actually associates with PRC2 (and that the lack of function is not simply due to lack of binding), the authors should employ a non-crosslinking method. For example, performing an RNA Immunoprecipitation (RIP) from nuclear fractions or chromatin-enriched fractions would be appropriate to verify if TERRAmut co-precipitates with PRC2 when tethered by dCas9.
(2) It is unclear whether the dCas9-sgRNA "tail" effectively competes with chromatin for PRC2 binding in the cellular context. The authors should experimentally demonstrate the competitive localization of PRC2 between the sgRNA decoy and its chromatin targets to clarify the mechanism of action. A ChIP-qPCR experiment at the CDKN2A locus in the presence of the dCas9-TERRAmut construct would clarify if PRC2 is displaced from nucleosomes, as suggested by the in vitro model.

2. The authors propose a dynamic equilibrium between monomeric and dimeric states driven by RNA sequence. However, the EMSA data (Extended Data Fig. 1) and the competition assay (Fig. 2e) display discrete bands without the intermediate shifts or smearing expected from a dynamic equilibrium of species with such distinct masses (~350 vs ~700 kDa). Relying on standard SEC retention volume (Fig. 2c) or affinity-grid EM (Extended Data Fig. 2) is insufficient to claim absolute molecular weight in solution, as these methods are influenced by particle shape and grid interactions. The authors whould rather perform SEC-MALS (Multi-Angle Light Scattering) to definitively measure the absolute molecular weight and stoichiometry of the Pyrimidine-rich vs. G-rich RNA-bound complexes in solution.

3. The authors propose a model where G-rich RNAs (including TERRAmut) induce PRC2 dimerization by bridging two protomers, likely engaging the flexible loops of the EZH2 subunit (as suggested in the Cryo-EM section and Extended Data Fig. 5). While the authors validated the role of AEBP2 for monomeric binding (Fig. 3c), they did not validate the protein interface responsible for dimerization. If the "flexible loops" are indeed the key to this G-rich RNA recognition and subsequent dimerization, mutations of positively charged residues within these loops (e.g., EZH2 CXC domain or N-terminal disordered regions) should abolish dimerization and restore methyltransferase activity in vitro, even in the presence of TERRAmut. The authors should generate specific point mutants or domain deletions (guided by their Cryo-EM or AlphaFold models) and perform EMSA and methyltransferase assays to prove that these specific residues are essential for RNA-mediated dimerization and inhibition.

4. The manuscript treats G-rich and Pyrimidine-rich RNAs as binary entities. However, physiological lncRNAs (e.g., XIST, HOTAIR) are long and contain mixed sequences. It is unclear which binding mode prevails when a single RNA molecule contains both G-rich and Pyrimidine-rich motifs. Does the high-affinity monomeric binding (Pyrimidine-rich/AEBP2) prevent the dimerization (G-rich/EZH2), or does the dimerization motif dominate and force the complex into an inhibited state? To address biological relevance, the authors should design a chimeric RNA construct containing both a Pyrimidine-rich tract (e.g., Poly(C) or P10) and a G-rich tract (e.g., TERRAmut) separated by a linker. Testing this RNA in EMSA (to check for monomer vs. dimer) and activity assays would reveal the hierarchy of these regulatory elements.

5. The FRET assay is key to the argument that different RNAs bind independent sites. However, the current presentation (Fig. 2d) lacks sufficient controls. Please provide a detailed schematic illustrating the positioning of the fluorophores and the hypothesized complex assembly. In addition, to rule out non-specific proximity effects or dye-RNA interactions, a negative control using a non-structured, non-binding RNA (e.g., Poly(A)) must be included under identical conditions.

6. The authors assert that AEBP2 is the primary driver for Pyrimidine-rich RNA binding (Fig. 3). However, the data in Figure 1 shows that the 5-subunit complex (containing AEBP2 but lacking JARID2) has reduced affinity for Pyrimidine-rich RNAs compared to the 6-subunit complex. This suggests that JARID2 also contributes significantly to binding Pyrimidine-rich RNAs. Focusing solely on AEBP2 (Fig. 3c) might oversimplify the mechanism. The authors should clarify the interplay

between JARID2 and AEBP2. Does the AEBP2Δ261–389 mutant completely abolish Pyrimidine-rich binding in the absence of JARID2? Or does JARID2 provide a redundant binding surface? Comparing the RNA binding of the "5-subunit AEBP2-mutant" vs "6-subunit AEBP2-mutant" would disentangle the contributions of these two accessory subunits.

7. To strengthen the comparative analysis between WT PRC2 and the AEBP2Δ261–389 mutant, it is not enough to simply list Kd values derived from separate experiments. For a robust assessment of how the mutation alters RNA binding preferences, the authors should present binding profiles for both WT and mutant proteins on the same gel to eliminate inter-assay variability and allow for direct visual comparison of the gel shifts.
Major concerns:
1. The triangle indicating serial dilution is oriented opposite to the intuitive flow seen in other figures. Standardizing this would improve readability.
2. In the Abstract, the sentence "PRC2 binds many different RNAs with similar affinity, however, the functional effect on enzymatic activity depends entirely on the sequence..." contains a comma splice. It should be corrected to: "PRC2 binds many different RNAs with similar affinity; however, the functional effect..." or split into two sentences.
3. Throughout the manuscript, the spacing is inconsistent. It appears as "AlphaFold3" and "AlphaFold 3". Please standardize the usage.
4. The manuscript alternates between "CLIPseq" (Abstract) and "CLIP-seq". Please use a consistent format, preferably "CLIP-seq" or "HITS-CLIP" as per field standards.
5. There appears to be a blurred line or masking artifact at the bottom of Figure 2. Please ensure the image quality is corrected.
6. Latin terms such as in vitro, in vivo, de novo, cis, and trans should be italicized throughout the text.
7. On Page 10, under AUTHOR CONTRIBUTIONS, there is a typo in the sentence: "performed biochemiscal experiments." This should be corrected to "biochemical".
8. On Page 3, Paragraph 3, the sentence begins with "Alternatively, the six-subunit complex binds RNA sequences and structures more broadly." The use of "Alternatively" is confusing here because this statement does not present an alternative to the previous sentence (which discussed the 4-subunit complex preferring G4), but rather contrasts the behavior of the 6-subunit complex. A transition like "In contrast," or "Conversely," would improve logical flow.
9. In the legend for Figure 4d, the text reads "Affinity (Kd): PRC2-RNA binding affinities measured by EMSA." It would be more precise to state "Representative Kd values derived from EMSA" or simply "Binding affinity (Kd)..." to avoid redundancy. Also, ensure statistical symbols like P (value) and t (test) are italicized in Figure 5 legend.
10. Throughout the Methods section, the manuscript inconsistently refers to chromatography columns and instruments. For example, "HiTrap Heparin HP column (Cytiva)" is used, while "Superose 6 increase 10/300 column (GE Healthcare)" is used. Similarly, the Typhoon phosphorimager is attributed to GE Healthcare. Given that GE Healthcare Life Sciences was rebranded as Cytiva in 2020, the authors should update these trade names to be consistent or clarify if legacy equipment is being used.
11. In the "HEK 293T/17 cell culture" and "G-401 cell culture" sections, the temperature is written as "37 degree." This should be corrected to the standard scientific notation "37°C."
12. In the same sections, "CO2" is written without a subscript. It should be formatted as "CO_2."
13. On Page 14 (EMSA section) and Page 16 (Methyltransferase assay), the text mentions "Prism software" for data fitting. The authors should specify the manufacturer (GraphPad) and the version number (e.g., GraphPad Prism 9) used for analysis. Similarly, for "ImageQuant," the version number should be provided.
14. In the "Methyltransferase activity assay" section, the regression equation is described as "log(inhibitor) vs. response -- variable slop". This is a typographical error and should be corrected to "variable slope."
15. In the "Analytic size-exclusion chromatography" section, the manufacturer is listed as "Bio-rad". The correct capitalization is "Bio-Rad."

Version 1:

Reviewer comments:

Reviewer #2

(Remarks to the Author)
The authors state that, following consultation with the editor, they have removed Figure 5 and the CRISPR–dCas9–based cellular experiments, which were the primary focus of my major concerns. As my original comments were largely centered on the interpretation and validation of these in vivo experiments, I do not have substantial additional comments on the revised manuscript. Nevertheless, I remain of the view that cellular validation is important, as the in vitro findings would benefit from confirmation in a cellular context. Without such evidence, the ongoing debate in the PRC2–RNA field may remain unresolved.

Reviewer #3

(Remarks to the Author)
Reviewer Comments to the Author

- Manuscript Title: RNA-induced PRC2 inhibition depends on the sequence of bound RNA
- Recommendation: Accept with Minor Revisions

- General Comments: I have reviewed the revised manuscript entitled "RNA-induced PRC2 inhibition depends on the sequence of bound RNA." The authors have satisfactorily addressed the majority of the concerns raised in the previous round of review. This manuscript presents a significant and timely advance in our understanding of the epigenetic regulator Polycomb Repressive Complex 2 (PRC2) and its modulation by RNA. While the inhibitory role of high-affinity G-quadruplex (G4) RNAs via PRC2 dimerization is established, the interaction between PRC2 and the wider transcriptome has remained elusive. The authors address this gap by demonstrating that the functional outcome of PRC2-RNA binding is strictly dictated by RNA sequence and secondary structure, rather than binding affinity alone. The study provides compelling evidence that the "dimerization-inhibition" mechanism extends beyond canonical G4 RNAs to include G-rich RNAs lacking consecutive guanosines and those forming pUG-folds. In a crucial contrast, the authors show that pyrimidine-rich RNAs (including cellular CLIP-seq motifs) bind PRC2 monomers without inducing dimerization, thereby retaining methyltransferase activity. This structural dissection—distinguishing between RNAs that compete for nucleosome binding via dimerization and those that do not—elegantly resolves apparent contradictions in the field regarding PRC2-RNA interactions. The experimental approach, combining cryo-EM structural insights with rigorous biochemical assays, supports the conclusion that RNA-mediated regulation of PRC2 is highly specific. This work not only clarifies the mechanism of PRC2 regulation but also suggests a broader paradigm where RNA sequence determines the functional state of chromatin-modifying complexes. The manuscript is of high quality and broad interest. I recommend publication in Nature Communications pending the resolution of the minor issues listed below.

- Minor Comments:
1. Contextual Bridge in the Introduction While the Introduction provides a solid background, the transition between the first and second paragraphs feels somewhat abrupt. I recommend bridging this gap with additional context regarding Ribonucleoprotein (RNP) binding modes and their general interaction dynamics.
> Reasoning: Providing a richer description here would not only make the manuscript more accessible to a broader readership but also highlight the study's relevance to general RNP biology. Consequently, this expansion would likely increase the manuscript’s visibility and citation potential among researchers studying RNP interactions beyond the specific PRC2 field.

2. Clarification on Mass Photometry (MP) Data and Oligomerization (Re: Previous Comment 2) Regarding the mechanism proposed, where poly-pyrimidine sequences support monomeric PRC2 (activation) and G4 sequences induce dimerization (inhibition), I find the response to the previous "Comment 2 of Reviewer #3" concerning the Mass Photometry (MP) results to be partially insufficient.
> Specific Concern: The authors argue for G4-mediated dimerization; however, considering the reported Kd values, one would theoretically expect a significantly higher proportion of the dimeric form in the PRC2-TERRA complex than what is currently presented in the MP histograms.
> Suggestion: Please explicitly address why the MP results might show a lower-than-expected dimer population compared to what the affinity data (Kd) suggests. If this is a technical limitation of the MP assay under these specific conditions or a dynamic equilibrium issue, it should be discussed to ensure the core thesis regarding G4-induced dimerization remains robust.
Conclusion: This manuscript represents a valuable contribution to the field of epigenetics and RNA biology. I look forward to seeing the final version with these minor textual and interpretive clarifications.

Open Access This Peer Review File is licensed under a Creative Commons Attribution 4.0 International License, which

permits use, sharing, adaptation, distribution and reproduction in any medium or format, as long as you give appropriate credit to the original author(s) and the source, provide a link to the Creative Commons license, and indicate if changes were made.
In cases where reviewers are anonymous, credit should be given to 'Anonymous Referee' and the source.
The images or other third party material in this Peer Review File are included in the article's Creative Commons license, unless indicated otherwise in a credit line to the material. If material is not included in the article's Creative Commons license and your intended use is not permitted by statutory regulation or exceeds the permitted use, you will need to obtain permission directly from the copyright holder.
To view a copy of this license, visit https://creativecommons.org/licenses/by/4.0/

RESPONSE TO REVIEWER COMMENTS

Reviewer #1 (Remarks to the Author):

In this manuscript Song et al. continue their work on structurally and mechanically characterising the regulation of the histone methyltransferase complex PRC2 by the interaction with RNAs. In their foundational paper [Song et al. 2023] they had described the structural basis of PRC2 regulation by G-quadruplex RNAs. Many of the RNAs PRC2 has been reported to interact with have however no canonical G-quadruplex sequences and/or structures. To address this conundrum the authors systematically test the interactions of a diverse set of RNA sequences with PRC2 using biochemical and structural methods (single particle (cryo)EM but also AlphaFold3 mediated structure prediction).
Their main findings are that PRC2 quiet promiscuously binds to many different RNA sequences and that RNAs without canonical G quadruplex structures can still induce the previously observed inhibitory PRC2 dimer formation. They also show conclusively that only RNA sequences which induce this PRC2 dimer have an inhibitory effect on its histone methyltransferase activity. RNAs that bind PRC2 with high affinity but do not induce dimer formation (e.g. pyrimidine rich sequences) do not inhibit its activity. These insights reconciles previous findings of apparent contradictory effects of different RNA sequences on PRC2 activity.
The study is thorough in its inquiry and the presented data, especially the EM data, are beautifully displayed, conclusive and carefully interpreted.
We appreciate the generous comments from Reviewer 1, especially with the recognition of our work's significance that '*These insights reconciles previous findings of apparent contradictory effects of different RNA sequences on PRC2 activity.*'

Minor concerns:
In the previously reported EM map of the G-quadruplex induced PRC2 dimer (EMD-29578; PDB:8FYH) the G-quadruplex appears to contact both PRC2 promoters. It is unclear to this reviewer wether this is also the case with their TERRAmut RNA here. Do the authors observe unexplained density which might be RNA related? If not is this potentially be an issue of a too tight map around the RNA density?
In the extended Figure 5 (now we moved this figure to main Figure 3b), we compared local resolution-filtered density map of TERRAmut-bound PRC2 with the model of G-quadruplex RNA-bound PRC2 and found that TERRAmut RNA binding, but not G-quadruplex RNA binding, leads to the visualization of additional density of RBAP48 or AEBP2 (black arrow). We explained this difference in the main text '*Also, the* $TERRA_{mut}$ *density is attached to the bottom lobe of one PRC2 protomer in a region of RBAP48 and AEBP2, which was less structured in the G4 RNA-bound map (Extended Data Fig. 5). This*

difference may indicate that the TERRA$_{mut}$, expected to be more extended than a G4, interacts with additional protein surfaces.'

In the supplementary Figure 4b, we present the Fourier shell correlation (FSC) curves of consensus map and two individual bodies in which consensus and body 1 include TERRAmut RNA. For all constructions, the corrected FSC curves overlap with the masked FSC curves, indicating that the refinement masks are not overly tight and that the reported resolutions are not overestimated.

Reviewer #2 (Remarks to the Author):

In this manuscript, Song et al. investigate how RNA sequence composition differentially regulates the activity of Polycomb Repressive Complex 2 (PRC2). Through a combination of biochemical assays, negative-stain and cryo-electron microscopy, AlphaFold3 modeling, and a CRISPR-dCas9–based RNA localization system, the authors demonstrate that RNA binding to PRC2 is functionally heterogeneous. Specifically, G-rich RNAs, including non-canonical sequences incapable of forming classical G-quadruplexes, induce PRC2 dimerization (AEBP2 and JARID2 included) and inhibit methyltransferase activity, whereas pyrimidine-rich RNAs bind PRC2 in a monomeric, catalytically competent state. The work provides a coherent mechanistic explanation reconciling disparate reports on PRC2-RNA interactions.

The work is technically strong, conceptually interesting, and timely. The authors convincingly demonstrate that PRC2 engages distinct classes of RNAs through mechanistically separable modes of interaction. It represents a substantial advance in our understanding of PRC2–RNA regulation and provides a coherent mechanistic framework that reconciles long-standing inconsistencies in the field. However, in light of the ongoing controversy surrounding PRC2–RNA interactions in cellular contexts, several aspects, particularly the scope and validation of the CRISPR-dCas9–based RNA localization assay, require clearer experimental demonstration to strengthen the manuscript for Nature Communications.
We appreciate the Reviewer 2's comments and useful suggestions which help to increase the clarity of experiments.

Major Comments

1. Requirement for solid validation using CRISPR-display, related to Figure 5

Although TERRAmut robustly induces PRC2 dimerization and inhibits catalytic activity in vitro, it does not elicit derepression of CDKN2A in the CRISPR-dCas9-based RNA localization assay. While the authors propose plausible explanations, such as reduced effective affinity or competition with endogenous RNA-binding proteins, this discrepancy underscores a key limitation of this cellular assay.
Given the growing number of recent reports describing context-dependent or divergent PRC2–RNA interactions in cells, the robustness and dynamic range of the CRISPR-dCas9-based RNA localization assay must be more clearly established. In this regard, authors should include TERRA WT as a positive control. Demonstrating PRC2 inhibition

and target gene derepression at the CDKN2A locus would substantially strengthen the cellular relevance of the conclusions.

We appreciate the reviewer’s emphasis on rigorous in vivo validation, particularly given the reported discrepancies regarding PRC2-RNA interactions. However, the proposed experiments are outside our current technical expertise and would require substantial time for optimization. In consultation with the editor, we have therefore removed Figure 5 and the related CRISPR-dCas9 content to avoid overinterpretation, and we now explicitly acknowledge the lack of in vivo validation as a limitation of this study in the Discussion.

2. In vivo (or ex vivo) relevance of RNA-induced PRC2 dimerization

A central conclusion of the manuscript is that RNA-induced PRC2 dimerization underlies sequence-dependent inhibition of PRC2 activity. While this mechanism is convincingly demonstrated in vitro, its prevalence and relevance ex vivo remain insufficiently established. The CRISPR-dCas9 experiments target a single locus (CDKN2A) in one cancer-derived cell line, making it difficult to assess whether RNA-mediated PRC2 inhibition represents a general regulatory mechanism or a locus-specific effect under artificial conditions. Extending these analyses to additional canonical PRC2 target loci, such as the HOXA cluster, would substantially strengthen the generality of the conclusions.

See response to comment 1.

3. Interplay between RNA and nucleosomal DNA binding at AEBP2 Zn finger and the R-rich motif

The authors show that an R-rich motif within AEBP2 contributes to RNA binding. This region has been previously well characterized as a nucleosomal DNA binding interface (PMIDs: 29681498, 33479123). The manuscript would benefit from a more comprehensive discussion integrating these findings, specifically addressing whether RNA and nucleosomal DNA binding are mutually exclusive, competitive, or context-dependent.

It would be of interest to determine whether pyrimidine-rich RNAs preferentially engage the Zn-finger domain while nucleosomal DNA interacts with the R-rich motif, or vice versa, thereby potentially cooperating to facilitate PRC2 recruitment. This possibility could be readily tested using electrophoretic mobility shift assays (EMSA).

In the revised manuscript, we performed DNA-RNA competition assays using six-subunit WT PRC2 and PRC2 AEBP2Δ379-389 in which only the arginine-rich motif of AEBP2 is deleted. We find that a 60-bp dsDNA substrate is efficiently displaced by the

G-rich TERRAmut RNA, but not by the pyrimidine-rich poly(C) RNA. This result is consistent with our nucleosome-RNA competition data, supporting the conclusion that only RNAs that induce PRC2 dimerization effectively antagonize PRC2-DNA binding. Notably, higher concentrations of TERRAmut are required to displace dsDNA from the $PRC2_{AEBP2\Delta 379\text{-}389}$ than from WT PRC2 (IC□□,TERRAmut($PRC2_{AEBP2\Delta 379\text{–}389}$) = 217 nM vs. IC□□,TERRAmut(WT) = 48 nM), consistent with reduced TERRAmut affinity upon deletion of this arginine-rich motif and supporting our model that this motif contributes to TERRAmut binding.

4. Based on the dimerization and PRC2 binding results, it would be informative to assess whether TERRAmut fails to induce dimerization of the four-subunit PRC2 core complex.

RNA-mediated dimerization is driven primarily by the catalytic subunit EZH2, through its arginine-rich loop and the CXC-CXC protein-protein dimer interface. Accordingly, we do not anticipate a substantial change in TERRAmut-induced dimer formation with the four-subunit core complex, although it would require higher RNA concentration because of the weaker affinity. Unfortunately, we do not currently have four-subunit complex to test this. The recombinant four-subunit core complex is experimentally challenging: it has been reported to dimerize in vitro independently of RNA (with a distinct dimer arrangement), and it exhibits substantial conformational flexibility in the region corresponding to the accessory-protein binding site (Davidovich et al., 2014, *Nucleic Acids Research)*, which is normally stabilized by accessory subunits.

In addition, multiple studies have shown that, in cells, PRC2 primarily exists as higher-order assemblies containing accessory factors (PRC2.1 and PRC2.2), rather than as the isolated four-subunit core. While the four-subunit core is sufficient for catalytic activity, it is likely less physiologically representative of endogenous PRC2 species.

Minor Comment

1. In this sentence, “We first prepared the six-subunit PRC2 complex with accessory proteins AEBP2 and JARID2, as these accessory proteins have previously been implicated in RNA binding…”, the authors should explicitly state that they used the short (embryonic) isoform of AEBP2 and a truncated form of JARID2. The precise transcript variant and amino-acid boundaries (e.g., aa xx–yy) should be clearly specified for consistency with the Materials and Methods as well.

We now include that information in both Results and Materials and Methods.

2. In this sentence, “Thus, our systematic characterizations indicate that the four-subunit holoenzyme contains all essential protein elements required to recognize G4 as the most preferred RNA, consistent with our cryo-EM structure, which shows that the catalytic subunit EZH2 is predominantly responsible for G4 RNA recognition.”, the authors should add the appropriate reference supporting the cryo-EM structural evidence.
We edited the sentence and added references.

3. **Figure 1a**: Including a simplified schematic or cartoon representation of the RNA secondary/tertiary structures would help readers more immediately grasp the rationale behind RNA selection. This does not need the level of detail shown in Extended Data Fig. 1a, but should clearly convey structural differences among the RNAs used.
We added suggested cartoon representation as Figure 1b.

4. Overall, the manuscript is occasionally difficult to follow, as essential information is dispersed across multiple sections and different PRC2 complexes are used in different experiments. In order to increase the readability, I suggest several points as follows.

(1) Clearly specify, both in the figure legends and at the first mention in the main text, which PRC2 complex was used for each experiment. For example, the authors should explicitly state when the six-subunit PRC2 complex was used for cryo-EM, size-exclusion chromatography, and related analyses.
This is an important suggestion, and we have edited the text in accordance.

(2) In the legend of Figure 5a, the authors should mention the cell line they used.
We removed Figure 5.

5. Statistical reporting: Exact numbers of replicates (n) should be more consistently reported in figure legends, particularly for biochemical assays.

We now include exact numbers of replicates in the figure legends.

Reviewer #3 (Remarks to the Author):

Review Comments:

This study by Song et al. investigates the structural and functional consequences of RNA binding to Polycomb Repressive Complex 2 (PRC2). Using Cryo-EM and biochemical assays, the authors identify two distinct binding modes: G-rich RNAs (including non-G4 sequences like TERRAmut) induce PRC2 dimerization and enzymatic inhibition, whereas pyrimidine-rich RNAs bind PRC2 monomers via the AEBP2 subunit without inhibiting activity. The authors attempt to extend these findings to a cellular context using a CRISPR-dCas9 display system. This work addresses a long-standing debate regarding the specificity and outcome of PRC2-RNA interactions, offering a potentially unifying structural model where functional outcomes (inhibition vs. recruitment) are sequence-dependent. However, a significant gap remains between the rigorous in vitro structural data and the in vivo functional assays. Specifically, the finding that TERRAmut induces potent inhibition in vitro but is completely inactive in cells (Fig. 5) challenges the physiological relevance of the proposed non-G4 dimerization mechanism. Furthermore, the exclusion of standard in vivo binding validation (based on the inefficiency of UV crosslinking shown in Extended Data Fig. 10) leaves the cellular mechanism unproven. While the structural insights are novel, substantial revision including rigorous biophysical validation and more robust cellular evidence is required to substantiate the biological conclusions to meet the need for the scope of publication in Nature Communications.
We appreciate Review 3's thorough reading of our manuscript. We have conducted some additional experiments as suggested.

Major concerns:
1. While the in vitro characterization is extensive, the in vivo evidence is sparse, relying heavily on the functional outcome of the CRISPR-dCas9 system without validating the intermediate steps. There is a notable contrast between the results in Figure 4b, where TERRAmut significantly inhibits PRC2, and Figure 5, where this inhibitory effect is abolished in cells. The authors attribute this to "lower binding affinity," but this remains speculative. To support the mechanistic conclusions within a reasonable revision timeline, the following are necessary:
(1) The authors argue in Extended Data Fig. 10 that UV crosslinking is inefficient for PRC2. However, the lack of in vivo binding data leaves a gap in the mechanism. To confirm that the dCas9-tethered TERRAmut actually associates with PRC2 (and that the lack of function is not simply due to lack of binding), the authors should employ a non-crosslinking method. For example, performing an RNA Immunoprecipitation (RIP) from

nuclear fractions or chromatin-enriched fractions would be appropriate to verify if TERRAmut co-precipitates with PRC2 when tethered by dCas9.
(2) It is unclear whether the dCas9-sgRNA "tail" effectively competes with chromatin for PRC2 binding in the cellular context. The authors should experimentally demonstrate the competitive localization of PRC2 between the sgRNA decoy and its chromatin targets to clarify the mechanism of action. A ChIP-qPCR experiment at the CDKN2A locus in the presence of the dCas9-TERRAmut construct would clarify if PRC2 is displaced from nucleosomes, as suggested by the in vitro model.
See response to Reviewer 2 comment 1.

2. The authors propose a dynamic equilibrium between monomeric and dimeric states driven by RNA sequence. However, the EMSA data (Extended Data Fig. 1) and the competition assay (Fig. 2e) display discrete bands without the intermediate shifts or smearing expected from a dynamic equilibrium of species with such distinct masses (~350 vs ~700 kDa). Relying on standard SEC retention volume (Fig. 2c) or affinity-grid EM (Extended Data Fig. 2) is insufficient to claim absolute molecular weight in solution, as these methods are influenced by particle shape and grid interactions. The authors whould rather perform SEC-MALS (Multi-Angle Light Scattering) to definitively measure the absolute molecular weight and stoichiometry of the Pyrimidine-rich vs. G-rich RNA-bound complexes in solution.
We appreciate the reviewer's concern, although our negative-stain EM, cryo-EM, and SEC analyses yield consistent results. To obtain an independent estimate of the absolute molecular weight of the complexes, we performed mass photometry (MP). We did not have sufficient material to carry out multiple SEC-MALS experiments across different RNA sequences.

Because MP measurements are performed at 10-20 nM complex concentration, which is well below the Kd values for most PRC2-RNA interactions and below the concentration range that supports robust PRC2 dimerization, we stabilized PRC2-RNA assemblies by mild crosslinking prior to MP. Specifically, PRC2-RNA complexes were assembled at 1 μM PRC2 and crosslinked with 0.1% glutaraldehyde on ice for 25 minutes, then diluted to 20 nM and measured immediately by MP. A caveat of this approach is that the PRC2-PRC2 dimer interface involves only a small number of residues relative to the overall protein surface (Fig. 3), and the RNA-bridged interface is not efficiently captured by glutaraldehyde because glutaraldehyde does not crosslink nucleic acids to proteins. Consequently, after dilution we observed only a small dimer population; nevertheless, the dimer fraction was reproducibly seen for G-rich RNA-PRC2 complexes (see below). For poly(C) there was no more material at the dimer position than for the non-binding poly(A) control or for PRC2 alone.

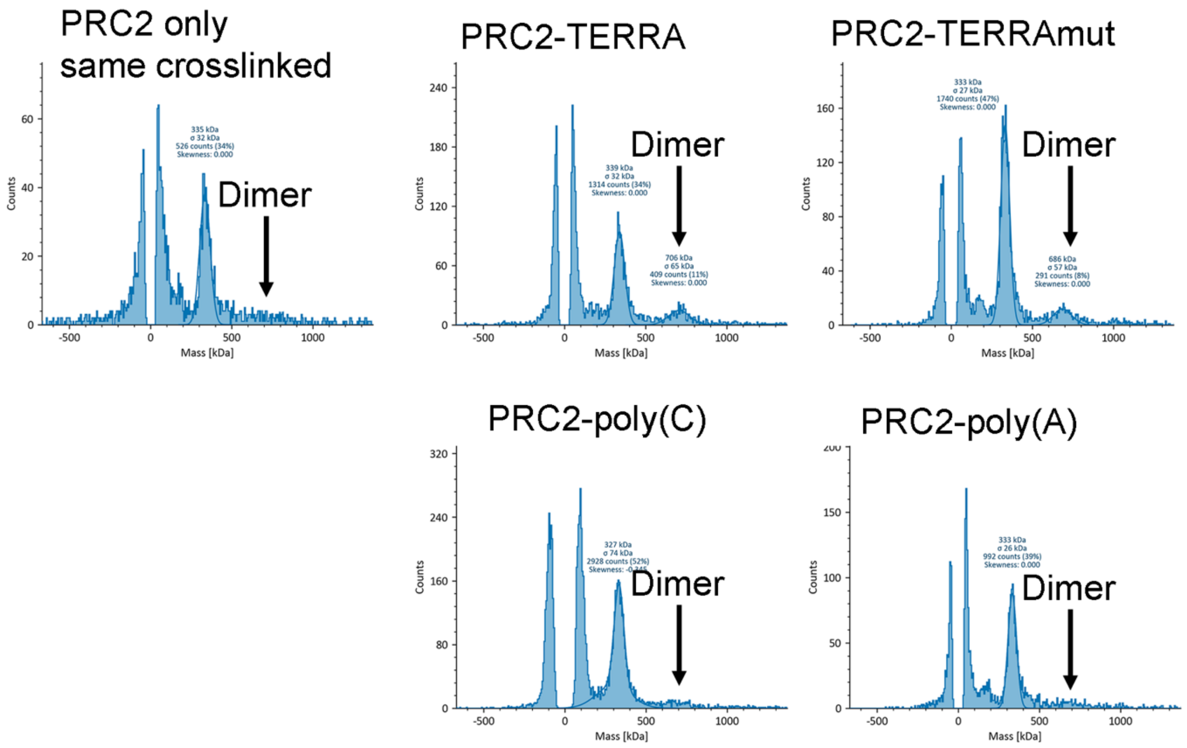

We repeated this experiment using another batch of PRC2 protein with the similar result.

3. The authors propose a model where G-rich RNAs (including TERRAmut) induce PRC2 dimerization by bridging two protomers, likely engaging the flexible loops of the EZH2 subunit (as suggested in the Cryo-EM section and Extended Data Fig. 5). While the authors validated the role of AEBP2 for monomeric binding (Fig. 3c), they did not validate the protein interface responsible for dimerization. If the "flexible loops" are indeed the key to this G-rich RNA recognition and subsequent dimerization, mutations of positively charged residues within these loops (e.g., EZH2 CXC domain or N-terminal disordered regions) should abolish dimerization and restore methyltransferase activity in vitro, even in the presence of TERRAmut. The authors should generate specific point mutants or domain deletions (guided by their Cryo-EM or AlphaFold models) and perform EMSA and methyltransferase assays to prove that these specific residues are essential for RNA-mediated dimerization and inhibition.

When we first reported G-quadruplex RNA-mediated PRC2 dimerization (PubMed 37733873), we tested multiple EZH2 mutants as suggested by the reviewer. Substituting residues at the EZH2 CXC-CXC dimer interface with alanine reduced dimer formation in negative-stain EM, whereas substitution of the same residues with tyrosine increased PRC2 binding to G-quadruplex RNA. We interpret this enhancement as arising from aromatic stacking between tyrosine side chains that can stabilize the CXC

dimer interface. We also tested several mutations in the EZH2 arginine-rich flexible loop, including a charge-reversal variant behaving as a gain-of-function mutant that rescues zebrafish development.

Because the TERRAmut-induced PRC2 dimer adopts an overall protein-RNA architecture that is nearly identical to the G-quadruplex RNA-induced dimer, we expect these mutants to exhibit similar effects with TERRAmut. For this reason, we did not include these additional mutant analyses in the present manuscript.

4. The manuscript treats G-rich and Pyrimidine-rich RNAs as binary entities. However, physiological lncRNAs (e.g., XIST, HOTAIR) are long and contain mixed sequences. It is unclear which binding mode prevails when a single RNA molecule contains both G-rich and Pyrimidine-rich motifs. Does the high-affinity monomeric binding (Pyrimidine-rich/AEBP2) prevent the dimerization (G-rich/EZH2), or does the dimerization motif dominate and force the complex into an inhibited state? To address biological relevance, the authors should design a chimeric RNA construct containing both a Pyrimidine-rich tract (e.g., Poly(C) or P10) and a G-rich tract (e.g., TERRAmut) separated by a linker. Testing this RNA in EMSA (to check for monomer vs. dimer) and activity assays would reveal the hierarchy of these regulatory elements.

We showed that G-rich and pyrimidine-rich RNAs are not mutually exclusive in PRC2 binding, which is consistent with the reviewer's model that physiological lncRNAs can contain both types of elements. We therefore hypothesized that dimer-inducing motifs represent the functional regulatory element within longer, more complex RNAs, and that pyrimidine-rich segments within the same transcript would not impede the activity of the regulatory motif.

To test this idea, we designed a chimeric RNA as suggested by the reviewer. It contained both pyrimidine-rich and G-rich sequences (Supplementary Fig. 9; chimeric RNA: P10$(A)_{10}$TERRA). This chimeric RNA inhibited PRC2 methyltransferase activity at about two-fold higher concentration than the G-rich RNA alone.

5. The FRET assay is key to the argument that different RNAs bind independent sites. However, the current presentation (Fig. 2d) lacks sufficient controls. Please provide a detailed schematic illustrating the positioning of the fluorophores and the hypothesized complex assembly. In addition, to rule out non-specific proximity effects or dye-RNA interactions, a negative control using a non-structured, non-binding RNA (e.g., Poly(A)) must be included under identical conditions.

We cannot provide a detailed schematic of the PRC2-TERRA-Poly(C) ternary complex, because we do not have definitive evidence mapping the PRC2-Poly(C) interaction interface. As mentioned in the Results, we made extensive attempts to determine a cryo-EM structure of Poly(C)-bound PRC2 but were unable to obtain a high-quality reconstruction sufficient for defining Poly(C) binding regions.

We agree that a non-binding, non-structured RNA control is important to rule out non-specific proximity effects and potential dye-RNA interactions. Following the reviewer's suggestion, we have added a negative control using Cy3-Poly(A) combined with Cy5-Poly(C) under identical conditions in the revised Fig. 2d. We also attempted to obtain Cy5-Poly(A) to test the reciprocal pairing (Cy3-TERRA with Cy5-Poly(A)), but the Cy5-Poly(A) oligo has been repeatedly back-ordered. To avoid delaying resubmission, we therefore do not include the Cy5-Poly(A) in this revision.

6. The authors assert that AEBP2 is the primary driver for Pyrimidine-rich RNA binding (Fig. 3). However, the data in Figure 1 shows that the 5-subunit complex (containing AEBP2 but lacking JARID2) has reduced affinity for Pyrimidine-rich RNAs compared to the 6-subunit complex. This suggests that JARID2 also contributes significantly to binding Pyrimidine-rich RNAs. Focusing solely on AEBP2 (Fig. 3c) might oversimplify the mechanism. The authors should clarify the interplay between JARID2 and AEBP2. Does the AEBP2Δ261–389 mutant completely abolish Pyrimidine-rich binding in the absence of JARID2? Or does JARID2 provide a redundant binding surface? Comparing the RNA binding of the "5-subunit AEBP2-mutant" vs "6-subunit AEBP2-mutant" would disentangle the contributions of these two accessory subunits.
We appreciate the reviewer's comment and agree that the higher affinity of the six-subunit complex relative to the five-subunit complex indicates that JARID2 can contribute to pyrimidine-rich RNA binding. We have revised the Discussion to clarify that pyrimidine-rich RNA recognition is supported by accessory subunits in combination, and that our data do not imply AEBP2 is the only contributor.

Our main conclusion from Fig. 3 (now Fig 4) is that the AEBP2 arginine-rich region makes a major contribution to pyrimidine-rich RNA binding, as truncation of this segment (AEBP2Δ261-389) substantially reduces binding compared with WT. The difference between five-subunit and six-subunit complexes in Fig. 1 is consistent with an additional, JARID2-dependent contribution that can further enhance overall affinity and/or stabilize the PRC2-RNA complex.

We agree that comparing "five-subunit AEBP2-mutant" and "six-subunit AEBP2-mutant" would help further disentangle these contributions. However, generating and validating the five-subunit AEBP2-mutant complex is experimentally demanding and would require extensive cloning, insect cell expression, and complex purification to obtain sufficient

material for quantitative binding assays. This is not feasible within the scope of this revision.

7. To strengthen the comparative analysis between WT PRC2 and the AEBP2Δ261–389 mutant, it is not enough to simply list Kd values derived from separate experiments. For a robust assessment of how the mutation alters RNA binding preferences, the authors should present binding profiles for both WT and mutant proteins on the same gel to eliminate inter-assay variability and allow for direct visual comparison of the gel shifts.
Please note that the Kd values we report already incorporate inter-assay variability because each EMSA dataset reflects multiple independent experiments performed on different days by at least two researchers and using at least two independent protein preparations (WT PRC2: L.Y., A.R.G., and V.T., n= 3 to 6; $PRC2_{AEBP2\Delta261\text{-}389}$: J.S. and A.R.G., n = 3). Accordingly, the reported mean ± standard deviation explicitly captures day-to-day and operator-to-operator variability.

Importantly, internal reproducibility is also supported by the observation that WT and $PRC2_{AEBP2\Delta261\text{-}389}$ display similar affinities for G-quadruplex RNAs (TERRA and pUG-fold), as expected. In contrast, TERRAmut shows a reproducible reduction in binding to $PRC2_{AEBP2\Delta261\text{-}389}$ relative to WT, consistent with our conclusion that the AEBP2 arginine-rich region contributes to TERRAmut binding.

Because of the robustness of our binding data and the limited availability of the recombinant mutant complex, we decline the suggestion for additional experiments.

Major concerns:
1. The triangle indicating serial dilution is oriented opposite to the intuitive flow seen in other figures. Standardizing this would improve readability.
We flipped Figure 2e.

2. In the Abstract, the sentence "PRC2 binds many different RNAs with similar affinity, however, the functional effect on enzymatic activity depends entirely on the sequence..." contains a comma splice. It should be corrected to: "PRC2 binds many different RNAs with similar affinity; however, the functional effect..." or split into two sentences.
We made this change.

3. Throughout the manuscript, the spacing is inconsistent. It appears as "AlphaFold3" and "AlphaFold 3". Please standardize the usage.
We now have ‘AlphaFold 3’ through the manuscript.

4. The manuscript alternates between "CLIPseq" (Abstract) and "CLIP-seq". Please use a consistent format, preferably "CLIP-seq" or "HITS-CLIP" as per field standards.

We made changes as suggested.

5. There appears to be a blurred line or masking artifact at the bottom of Figure 2. Please ensure the image quality is corrected.
Additional trimming was done for Figure 2 to remove the blurred line.

6. Latin terms such as in vitro, in vivo, de novo, cis, and trans should be italicized throughout the text.
Nature Portfolio suggests that those common Latin terms should be set in roman type, not italics.

7. On Page 10, under AUTHOR CONTRIBUTIONS, there is a typo in the sentence: "performed biochemiscal experiments." This should be corrected to "biochemical".
We edited it.

8. On Page 3, Paragraph 3, the sentence begins with "Alternatively, the six-subunit complex binds RNA sequences and structures more broadly." The use of "Alternatively" is confusing here because this statement does not present an alternative to the previous sentence (which discussed the 4-subunit complex preferring G4), but rather contrasts the behavior of the 6-subunit complex. A transition like "In contrast," or "Conversely," would improve logical flow.
We made the corresponding change.

9. In the legend for Figure 4d, the text reads "Affinity (Kd): PRC2-RNA binding affinities measured by EMSA." It would be more precise to state "Representative Kd values derived from EMSA" or simply "Binding affinity (Kd)..." to avoid redundancy. Also, ensure statistical symbols like P (value) and t (test) are italicized in Figure 5 legend.
We edited Figure 4d legend (now Figure 5d) and removed Figure 5.

10. Throughout the Methods section, the manuscript inconsistently refers to chromatography columns and instruments. For example, "HiTrap Heparin HP column (Cytiva)" is used, while "Superose 6 increase 10/300 column (GE Healthcare)" is used. Similarly, the Typhoon phosphorimager is attributed to GE Healthcare. Given that GE Healthcare Life Sciences was rebranded as Cytiva in 2020, the authors should update these trade names to be consistent or clarify if legacy equipment is being used.
We updated the Method as suggested.

11. In the "HEK 293T/17 cell culture" and "G-401 cell culture" sections, the temperature is written as "37 degree." This should be corrected to the standard scientific notation "37°C."

We removed this content.

12. In the same sections, "CO2" is written without a subscript. It should be formatted as "CO_2."
We removed this content

13. On Page 14 (EMSA section) and Page 16 (Methyltransferase assay), the text mentions "Prism software" for data fitting. The authors should specify the manufacturer (GraphPad) and the version number (e.g., GraphPad Prism 9) used for analysis. Similarly, for "ImageQuant," the version number should be provided.
We made suggested changes for both Prism and ImageQuant.

14. In the "Methyltransferase activity assay" section, the regression equation is described as "log(inhibitor) vs. response -- variable slop". This is a typographical error and should be corrected to "variable slope."
We corrected it.

15. In the "Analytic size-exclusion chromatography" section, the manufacturer is listed as "Bio-rad". The correct capitalization is "Bio-Rad."
We corrected it.

Response to reviewers' comments **March 27, 2026**

Reviewer #2 (Remarks to the Author):

The authors state that, following consultation with the editor, they have removed Figure 5 and the CRISPR–dCas9–based cellular experiments, which were the primary focus of my major concerns. As my original comments were largely centered on the interpretation and validation of these in vivo experiments, I do not have substantial additional comments on the revised manuscript. Nevertheless, I remain of the view that cellular validation is important, as the in vitro findings would benefit from confirmation in a cellular context. Without such evidence, the ongoing debate in the PRC2–RNA field may remain unresolved.
We thank the reviewer for this comment. This study focuses on the biochemical and structural basis of RNA sequence-dependent PRC2 regulation. We emphasize in the Discussion that cellular testing of these mechanisms is an important next step.

Reviewer #3 (Remarks to the Author):

Reviewer Comments to the Author

Manuscript Title: RNA-induced PRC2 inhibition depends on the sequence of bound RNA
Recommendation: Accept with Minor Revisions

General Comments: I have reviewed the revised manuscript entitled "RNA-induced PRC2 inhibition depends on the sequence of bound RNA." The authors have satisfactorily addressed the majority of the concerns raised in the previous round of review. This manuscript presents a significant and timely advance in our understanding of the epigenetic regulator Polycomb Repressive Complex 2 (PRC2) and its modulation by RNA. While the inhibitory role of high-affinity G-quadruplex (G4) RNAs via PRC2 dimerization is established, the interaction between PRC2 and the wider transcriptome has remained elusive. The authors address this gap by demonstrating that the functional outcome of PRC2-RNA binding is strictly dictated by RNA sequence and secondary structure, rather than binding affinity alone. The study provides compelling evidence that the "dimerization-inhibition" mechanism extends beyond canonical G4 RNAs to include G-rich RNAs lacking consecutive guanosines and those forming pUG-folds. In a crucial contrast, the authors show that pyrimidine-rich RNAs (including cellular CLIP-seq motifs) bind PRC2 monomers without inducing dimerization, thereby retaining methyltransferase activity. This structural dissection—distinguishing between RNAs that compete for nucleosome binding via dimerization and those that do not—

elegantly resolves apparent contradictions in the field regarding PRC2-RNA interactions. The experimental approach, combining cryo-EM structural insights with rigorous biochemical assays, supports the conclusion that RNA-mediated regulation of PRC2 is highly specific. This work not only clarifies the mechanism of PRC2 regulation but also suggests a broader paradigm where RNA sequence determines the functional state of chromatin-modifying complexes. The manuscript is of high quality and broad interest. I recommend publication in Nature Communications pending the resolution of the minor issues listed below.
We thank the reviewer for this positive assessment of our work and for recognizing the significance of our findings. We have addressed the minor issues raised below.

-Minor Comments:
1. Contextual Bridge in the Introduction While the Introduction provides a solid background, the transition between the first and second paragraphs feels somewhat abrupt. I recommend bridging this gap with additional context regarding Ribonucleoprotein (RNP) binding modes and their general interaction dynamics.
>Reasoning: Providing a richer description here would not only make the manuscript more accessible to a broader readership but also highlight the study's relevance to general RNP biology. Consequently, this expansion would likely increase the manuscript's visibility and citation potential among researchers studying RNP interactions beyond the specific PRC2 field.
We thank the reviewer for this suggestion. We have revised the Introduction to improve the transition by adding context on general non-canonical RNA-binding proteins and how distinct RNA-protein interactions can lead to different outcomes.

2. Clarification on Mass Photometry (MP) Data and Oligomerization (Re: Previous Comment 2) Regarding the mechanism proposed, where poly-pyrimidine sequences support monomeric PRC2 (activation) and G4 sequences induce dimerization (inhibition), I find the response to the previous "Comment 2 of Reviewer #3" concerning the Mass Photometry (MP) results to be partially insufficient.
> Specific Concern: The authors argue for G4-mediated dimerization; however, considering the reported Kd values, one would theoretically expect a significantly higher proportion of the dimeric form in the PRC2-TERRA complex than what is currently presented in the MP histograms.
> Suggestion: Please explicitly address why the MP results might show a lower-than-expected dimer population compared to what the affinity data (Kd) suggests. If this is a technical limitation of the MP assay under these specific conditions or a dynamic

equilibrium issue, it should be discussed to ensure the core thesis regarding G4-induced dimerization remains robust.

We agree that the MP data have limitations and should be interpreted cautiously. We therefore include the MP results in the response to the reviewers' comments as supporting evidence, rather than in the manuscript. Instead, our conclusions about dimerization are supported in the manuscript by two independent assays, size-exclusion chromatography and electron microscopy, both of which provide consistent evidence for PRC2 dimer formation in the presence of G-rich RNAs but not pyrimidine-rich RNAs. We note that the MP assay required crosslinking prior to measurement to preserve dimers during dilution to the low concentrations used for analysis, and this treatment could perturb RNA-binding ability of PRC2 and alter the binding equilibrium. In addition, because the dimer interface is relatively small compared with the overall size of PRC2, crosslinking may not efficiently capture and preserve the dimeric PRC2. For these reasons, MP may not provide robust detection of the dimer population under these conditions. We therefore view the MP results as supportive rather than primary evidence.

Conclusion: This manuscript represents a valuable contribution to the field of epigenetics and RNA biology. I look forward to seeing the final version with these minor textual and interpretive clarifications.

Reviewer Comments to the Author

Manuscript Title: RNA-induced PRC2 inhibition depends on the sequence of bound RNA **Recommendation:** Accept with Minor Revisions

General Comments: I have reviewed the revised manuscript entitled "RNA-induced PRC2 inhibition depends on the sequence of bound RNA." The authors have satisfactorily addressed the majority of the concerns raised in the previous round of review. This manuscript presents a significant and timely advance in our understanding of the epigenetic regulator Polycomb Repressive Complex 2 (PRC2) and its modulation by RNA. While the inhibitory role of high-affinity G-quadruplex (G4) RNAs via PRC2 dimerization is established, the interaction between PRC2 and the wider transcriptome has remained elusive. The authors address this gap by demonstrating that the functional outcome of PRC2-RNA binding is strictly dictated by RNA sequence and secondary structure, rather than binding affinity alone. The study provides compelling evidence that the "dimerization-inhibition" mechanism extends beyond canonical G4 RNAs to include G-rich RNAs lacking consecutive guanosines and those forming pUG-folds. In a crucial contrast, the authors show that pyrimidine-rich RNAs (including cellular CLIP-seq motifs) bind PRC2 monomers without inducing dimerization, thereby retaining methyltransferase activity. This structural dissection—distinguishing between RNAs that compete for nucleosome binding via dimerization and those that do not—elegantly resolves apparent contradictions in the field regarding PRC2-RNA interactions. The experimental approach, combining cryo-EM structural insights with rigorous biochemical assays, supports the conclusion that RNA-mediated regulation of PRC2 is highly specific. This work not only clarifies the mechanism of PRC2 regulation but also suggests a broader paradigm where RNA sequence determines the functional state of chromatin-modifying complexes. The manuscript is of high quality and broad interest. I recommend publication in *Nature Communications* pending the resolution of the minor issues listed below.

Minor Comments:

1. Contextual Bridge in the Introduction While the Introduction provides a solid background, the transition between the first and second paragraphs feels somewhat abrupt. I recommend bridging this gap with additional context regarding Ribonucleoprotein (RNP) binding modes and their general interaction dynamics.

- **Reasoning:** Providing a richer description here would not only make the manuscript more accessible to a broader readership but also highlight the study's relevance to general RNP biology. Consequently, this expansion would likely increase the manuscript's visibility and citation potential among researchers studying RNP interactions beyond the specific PRC2 field.

2. Clarification on Mass Photometry (MP) Data and Oligomerization (Re: Previous Comment 2) Regarding the mechanism proposed, where poly-pyrimidine sequences support monomeric PRC2 (activation) and G4 sequences induce dimerization (inhibition), I find the response to the previous "Comment 2 of Reviewer #3" concerning the Mass Photometry (MP) results to be partially insufficient.

- **Specific Concern:** The authors argue for G4-mediated dimerization; however, considering the reported K_d values, one would theoretically expect a significantly higher proportion of the dimeric form in the PRC2-TERRA complex than what is currently presented in the MP histograms.

- **Suggestion:** Please explicitly address why the MP results might show a lower-than-expected dimer population compared to what the affinity data (K_d) suggests. If this is a technical limitation of the MP assay under these specific conditions or a dynamic

equilibrium issue, it should be discussed to ensure the core thesis regarding G4-induced dimerization remains robust.

Conclusion: This manuscript represents a valuable contribution to the field of epigenetics and RNA biology. I look forward to seeing the final version with these minor textual and interpretive clarifications.
